# Removal of acid and basic dyes from textile wastewater using modified acrylic fibres waste as an efficient adsorbent

**DOI:** 10.1038/s41598-025-07134-y

**Published:** 2025-07-03

**Authors:** Thanaa F. Abdelhafez, Emad K. Radwan, Galal H. Sayed, Eman M. El Khatib, Mohamed F. Nasr, Lamiaa K. El Gabry

**Affiliations:** 1 Proteinic and Man-Made Fibres Department, Textile Research and Technology Institute, National Research Centre, Dokki, Giza, 12611 Egypt; 2 Water Pollution Research Department, National Research Centre, 33 El Buhouth St, Dokki, Giza, 12622 Egypt; 3 Faculty of Science, Ain Shams University, Cairo, 12622 Egypt

**Keywords:** Acrylic fibres waste, Sodium ethoxide, Dyes removal, Adsorption, Kinetic and isotherm studies, Environmental sciences, Chemistry

## Abstract

Acrylic fibre waste (AFW) was modified using sodium hydroxide and sodium ethoxide to improve its dye removal capabilities in textile wastewater. The optimization of modification conditions such as time, temperature, pH, and concentration was conducted to maximize adsorption efficiency. Fourier transform infrared spectroscopy (FTIR), field emission scanning electron microscope (SEM) and surface area measurement (BET) were utilized to characterize the unmodified and modified AFW. The AFW modified with Na-ethoxide and pure NaOH or NaOH mixture exhibited more pronounced cracks and pores compared to the unmodified fibres. FTIR analysis show that the conversion of the nitrile group of the unmodified AFW to an amide group after hydrolysis process. The modified AFW samples were tested for the removal all both dyes C.I. Acid Red 182 (AR182) and C.I. Basic Blue 9- methylene blue (MB) from wastewater. The results inducted that the removal percentage of approximately 96% for MB and 45% for AR182 dye at room temperature after 1 h. It was observed that the efficiency of dye removal was higher at lower temperatures. Isotherm studies, including Langmuir and Freundlich models, as well as kinetic studies, were conducted to analyze the adsorption process. The thermodynamic evaluation was estimated.

## Introduction

Synthetic organic dyes are artificial compounds created to add colour to different materials, including textiles, plastics, papers, and food products^1,2^. The textile industry utilizes synthetic organic dyes to dye fabrics, yarns, and fibers, enabling the production of colorful and patterned textiles^3^. However, synthetic organic dyes are toxic and non-biodegradable compounds. When these dye are released into natural water sources, synthetic dyes hander light penetration, disrupt the balance of aquatic organisms, and degrade the overall water quality^4,5^.

Many conventional and non-conventional treatment methods have been used for dye removal from contaminated water. Adsorption is a non-conventional method in which pollutants can be adsorbed onto the surface of the adsorbent material^6–8^.

The efficiency of the adsorption process for removing contaminants depends on two aspects: the characteristics of adsorbents and the conditions of the adsorption process^9^. Acrylic fiber has garnered significant attention over conventional adsorbents due to its high surface area, diverse range of porosity, and surface chemistry^10,11^. Labena et al.^12^ prepared adsorbent material from grafted acrylic membranes by using acrylic fibers waste applying phenylenediamine for the adsorption of Congo red and methylene blue dyes^12^. Acrylic fibres have extensive use in various applications, especially in eliminating of different contaminants like dyes, heavy metals, and pesticides. Many chemical modifications of acrylic fiber such as amination, reduction, and hydrolysis have been conducted to impart its ability to adsorb (anionic) acid, and reactive dyes. The alkaline hydrolysis of acrylic fiber transforms the fiber structure into an easily diffusible structure^13,14^. Alkaline hydrolysis leads to non-homogeneous and eroded surfaces to different degrees depending on the hydrolysis conditions^15–17^. Polyacrylonitrile fibers were chemically modified by conversion of their nitrile groups into other effective adsorbent groups under a two-step process. At first, the modification process was initiated through hydrolysis of the fibers in an alkaline solution. The second step, functionalization of the fibers was carried forward by thiourea. The thermogravimetric data clarified that the initial thermal stability of the modified fibers is lower than those of raw fibers due to conversion of nitrile groups into amine and thioamide functionalities^18^. Sodium hydroxide can hydrolyze the polyacrylonitrile fiber, changing the nitrile groups into carboxyl group. The surface of the polyacrylonitrile fiber was etched, resulting in changes of its performance including decrease in strength and increase in moisture regain. The study emphasizes the potential of hydrolysis agents, providing new insights into optimizing conditions for sustainable waste modification in the textile industry. Many studies have focused on the effect of processing on the physical, chemical and thermal properties of fibers as well as on utilizing acrylic fibers as dye removal material for different dyestuffs^19–22^.

Wastewater containing dyes is a well-known contaminant in industrial areas, impacting bodies of water. Due to their harmful effects on the environment, there has been significant emphasis on removing dyes from waste streams. Concerns have arisen regarding waste produced from acrylic fibers as they are non-biodegradable and release toxic gases, posing environmental threats. The research aims to repurpose acrylic fiber waste to create materials with improved dye absorption properties.

In this study, acrylic fiber waste (AFW) was modified using sodium hydroxide, sodium hydroxide in mixed solutions (water/solvent), and sodium ethoxide under different conditions to improve its adsorption capacity. The modified AFW was applied to remove basic and acid dyestuffs from aqueous solutions. Acid dye, C.I. Acid red 182 (AR182) and methylene blue C.I. Basic Blue 9 (MB) were selected as representatives for acidic and basic dyestuffs, respectively. Various factors affecting the adsorption process were studied, such as pH, dosage, contact time, initial concentration of dye, and temperature to determine the best conditions for dye removal. The pseudo-first-order and pseudo-second-order models were used to fit the adsorption kinetics while the Langmuir and Freundlich equations were used to fit the equilibrium isotherms. This new approach in using waste materials as adsorbent help eliminate other waste, significantly reducing costs. This new approach in using waste acrylic fiber materials to create technical adsorbent material to eliminate other waste, and significantly reducing costs.

## Methods

### Materials and chemicals

AFW was collected from local textile carpets and blankets companies. C.I. Basic blue 9 Methylene Blue (MB) and C.I. Acid Red 182 (AR182) dyes were purchased from Merck, Darmstadt, Germany. The molecular formula, molecular weight, and chemical structure of the dyes are given in Table 1. Sodium ethoxide (C_2_H_5_ONa) (Na-ethoxide) and dimethylformamide (DMF) were supplied by Sigma-Aldrich. Sodium hydroxide and absolute ethanol were supplied by El-Nasr Company for Fertilizers and Chemical Industries, Egypt. All other chemicals used are of purity higher than 99% and were used without any purification.

**Table 1:** Properties of the used synthetic organic dyes.

Color index	Molecular formula	Molecular weight	Chemical structure
C.I. acid red 182	C_16_H_13_N_3_O_4_S	343.36	
C.I. basic blue 9	C_16_H1_8_N_3_SCl	319.852	

### Modification of acrylic fibers waste

AFW underwent four different chemical modifications.

1. AFW was hydrolyzed with different concentrations of aqueous sodium hydroxide solution (0.1–1.0 M) at temperatures ranging from 60 to 95 °C for durations of 30–120 min using a material-to-liquor ratio (MLR) of 1:25.
2. AFW was modified with a binary mixture solution NaOH in (water /ethanol) at different ratios (90/10, 80/20, and 70/30) (NaOH/ethanol) containing 1.0 M NaOH at temperatures of ranging from 30 to 70 °C for 30–120 min with an MLR of 1:25.
3. AFW was modified with binary mixture solutions (water/ DMF) at different ratios (90/10, 80/20, and 70/30) (NaOH/ DMF) containing 1.0 M NaOH at temperatures ranging from (60–95) °C for 30–120 min.
4. AFW was modified with varying concentrations (0.3–1.0 M) of aqueous sodium ethoxide for 30, 45, and 60 min at temperatures ranging from 70 to 95 °C. After each modification, the samples were washed multiple times with running water and air-dried at room temperature.

### Characterization of modified AFW

#### Fourier transform Infrared spectra (FTIR)

Fourier transform infrared spectra were recorded on FTIR Nicolet 5 DX spectrophotometer using a wavelength range from 400 to 4000 cm^−1^. The samples of the unmodified and modified ACFW were examined as 1.5% KBr pellets.

#### Scanning electron microscope (SEM)

The morphology of the unmodified and modified AFW surface was characterized using scanning electron microscopy (SEM) of high resolution (Bruker Nano Gmb H Scanning Electron Microscope D-12489 Berlin, Germany).

#### Thermogravimetric analysis (TGA)

Thermal stability of the modified acrylic waste AFW was examined via using a SDT Q600V20.9 Build 20 instrument, firstly the samples were heated at the rate 10 °C/min from room temperature till 700 °C in an inert nitrogen atmosphere.

#### Surface area measurement (BET)

Brunauer–Emmett–Teller (BET) specific surface areas of unmodified acrylic fibre waste and modified acrylic fibre waste with Na-ethoxide were measured by N2 adsorption–desorption isotherm with a Quantachrome NOVA touch LX4 apparatus (Quantachrome Instruments, USA).

#### Dye removal studies

Stock solutions of MB and AR182 dyes were prepared by dissolving 0.1 g of each dye in 1000 mL of distilled water. Serial dilutions were subsequently performed to obtain the required concentrations throughout the study. The absorbance of the solutions of MB or AR182 dyes was measured using JENWAY-6405 UV/Vis spectrophotometer (Bibby Scientific LTD. UK) at wavelengths 655 nm and 488 nm, respectively. Single-component batch adsorption experiments were performed by immersing a weight of the unmodified or modified AFW in a fixed volume of a dye solution containing a known initial concentration in stoppered flasks. The flasks were shaken at 110 rpm and ambient temperature for 120 min. Samples were taken at different contact times and the remaining concentration of dyes was determined. The percentage of dye removal was calculated according to Eq. 1.

1 $${{R \% = }}\left( {\frac{{{\text{C}}_{{\text{o}}} {\text{ - C}}_{{\text{t}}} }}{{{\text{C}}_{{\text{o}}} }}} \right){100}$$

where C_o_ and C_t_ are the concentrations (mg/L) of the working solution and after a specific time of contact with the AFW, respectively.

The experiments were performed at different initial pH (pH_o_) values, different amounts of the modified AFW, and different temperatures to evaluate the influence of these factors on the adsorption process. The displayed data represents the average calculated from triplicate experiments. The pseudo-first-order (PFO)^23^, and pseudo-second-order (PSO)^24^ models were applied to the experimental kinetic results. Equation 2 and 3 give the non-linear forms of the PFO and PSO models, respectively.

2 $${\text{q}}_{{\text{t}}} {\text{ = q}}_{{\text{e}}} {\text{ (1 - e}}^{{{\text{ - k}}_{{1}} {\text{t}}}} {)}$$

3 $${\text{q}}_{{\text{t}}} { = }\frac{{{\text{k}}_{{2}} {\text{q}}_{{\text{e}}}^{{2}} {\text{t}}}}{{{\text{1 + k}}_{{2}} {\text{q}}_{{\text{e}}} {\text{t}}}}$$

where q_t_ and q_e_ (mg/g) are the amount of dye adsorbed per gram of the modified AFW at time t and at equilibrium state, respectively, k_1_ (1/min) and k_2_ (mg/g min) are the rate constants of the PFO and PSO models, respectively. The values of q_t_ and q_e_ were calculated by mass balance Eqs. 4 and 5.

4 $${\text{q}}_{{\text{t}}} { = }\frac{{\left( {{\text{C}}_{{\text{o}}} {\text{ - C}}_{{\text{t}}} } \right){\text{ V}}}}{{\text{W}}}$$

5 $${\text{q}}_{{\text{e}}} { = }\frac{{\left( {{\text{C}}_{{\text{o}}} {\text{ - C}}_{{\text{e}}} } \right){\text{ V}}}}{{\text{W}}}$$

where C_e_ (mg/L) is the concentration of dye in the solution at equilibrium state, W (g) is the mass of the modified AFW, and V (L) is the volume of the dye solution.

Adsorption isotherms depict the relationship between the adsorptive and adsorbent materials, explaining the equilibrium state between them. The assumptions of the best-fitting isotherm offer insights into both the surface properties and the adsorption mechanism^25^. Two isotherm models, the Freundlich and the Langmuir isotherms, were applied to explain the experimental equilibrium results. The Langmuir model assumes that the adsorption process takes place on homogeneous sites and monolayer^26^. Equation 6 represents the Langmuir model.

6 $${\text{q}}_{{\text{e}}} { = }\frac{{{\text{q}}_{{\text{m}}} {\text{k}}_{{\text{L}}} {\text{C}}_{{\text{e}}} }}{{{\text{1 + k}}_{{\text{L}}} {\text{C}}_{{\text{e}}} }}$$

where q_m_ (mg/g) is the theoretical maximum amount of dye per unit mass of adsorbent on the surface, and K_L_ (L/mg) is the Langmuir isotherm constant related to the affinity of the binding sites.

The Freundlich model assumes a heterogeneous surface and multilayer formation^27^. Equation 7 represents the non-linear form of the Freundlich model.

7 $${\text{q}}_{{\text{e}}} {\text{ = k}}_{{\text{f}}} {\text{ C}}_{{\text{e}}}^{{\text{1/n}}}$$

where K_F_ (mg^(1–1/n)^L^(1/n)^/g) is the Freundlich constant and 1/n (-) is a measure of the adsorption intensity.

The values of coefficient of determination (R^2^, Eq. 8) and chi-square (χ^2^, Eq. 9) were used as indicators for the quality of fit of the kinetic and isotherm models to the experimental data.

8 $$R^{2} = \frac{{\sum \left( {q_{{{\text{e}},{\text{cal}}}} - \overline{q}_{{{\text{e}},{\text{exp}}}} } \right)^{2} }}{{\sum \left( {q_{{{\text{e}},{\text{cal}}}} - \overline{q}_{{{\text{e}},{\text{exp}}}} } \right)^{2} - \sum \left( {q_{{{\text{e}},{\text{cal}}}} - q_{{{\text{e}},{\text{exp}}}} } \right)^{2} }}$$

9 $${\upchi }^{2} { } = { }\mathop \sum \limits_{{{\text{i }} = 1}}^{{\text{N}}} \left[ {\frac{{{ }\left( {{\text{q}}_{{{\text{e}},{\text{exp}}}} { } - {\text{ q}}_{{{\text{e}},{\text{cal}}}} } \right)^{2} }}{{{\text{q}}_{{{\text{e}},{\text{cal}}}} }}} \right]$$

where q_e,cal_, q_e,exp_ and ${\overline{\text{q}}}_{{{\text{e}},{\text{exp}}}}$ are the calculated, experimental, and average experimental amount of dye adsorbed by gram of the modified AFW at equilibrium, respectively, and N is the number of experimental points.

#### Thermodynamic studies

Adsorption thermodynamics and the nature of the adsorption process of methylene blue (MB) and Acid red 182 (AR182) were evaluated using three parameters: (i) change in free energy, the difference in Gibbs energy between the products and reactants (∆G^o^, KJ), (ii) enthalpy (∆H^o^, J), and (iii) entropy (∆S^o^, J/K). The parameters are essential in determining the feasibility and the nature of the adsorption process. They were determined using Eqs. 10 and 11^28^.

10 $$\Delta G^{o} = - RTlnK_{d}$$

11 $$\Delta G^{o} = \Delta H^{o} - T\Delta S^{o}$$

where K_d_ represents the dimensionless equilibrium constant, R (J/mol·K) is the gas constant, and T (K) denotes the absolute temperature. When a graph is plotted of lnK_d_ versus 1/T, it yields a straight line with a slope equal to − ΔH°/R and an intercept equal to ΔS°/R.

#### Reusability study

A reusability study was conducted to assess the adsorption capacity of modified acrylic fiber waste (AFW) over multiple adsorptions–desorption cycles using established methodologies with slight modifications^29^. The durability and regenerative potential of both unmodified and modified fibers were examined. A sample was exposed to repeated dye removal cycles in a solution containing 0.1 g/L methylene blue (MB), and the dye removal efficiency was measured using UV–visible spectroscopy. The reusability was determined by calculating the percentage of dye removal for each cycle. To ensure accurate determination of the removal percentage, the initial dye concentration of the solution was adjusted for each cycle by subtracting the amount of dye removed in the previous cycle.

## Results and discussion

### Fourier transform Infrared spectra (FTIR) of modification AFW

**Fig. 1:**
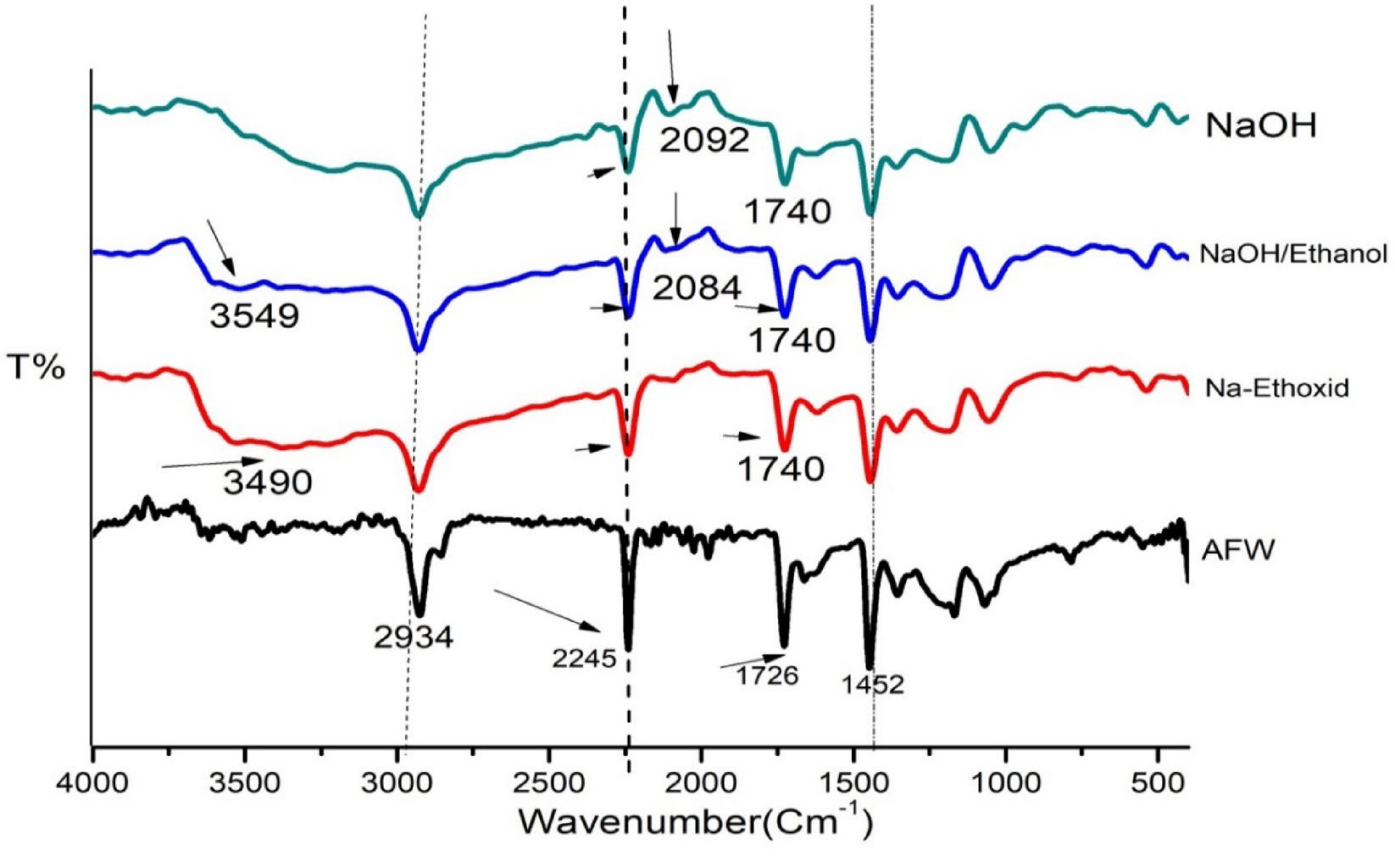
FTIR spectra of unmodified and modified ACFW. (Blank) unmodified AFW. (Red) 1 M Na-ethoxide, 95 °C, 1. (Blue) 0.5 M NaOH/ethanol) 50:50, 70 °C, 1 h. (Green) 1 M NaOH, 120 min, 95 °C, 1 h.

Figure 1 shows the Fourier Transform Infrared (FTIR) spectra of spectrum of all unmodified AFW as well as the modified AFW. The distinct peak at 2241 cm^−1^ corresponds to the nitrile (C≡N)^30^. The peaks at 1452 cm^−1^ and 1378 cm^−1^ are related to the bending vibration of CH_2_ and CH groups, while the peak at 1234 cm^−1^ is related to the stretching of C-H bonds in CH_3_ and CH_2_. The peak at 1738 cm^−1^ results from the C=O related to comonomers (such as methacrylate, styrene) present as acrylic additives. Notably, this peak exhibited a marked reduction in intensity across all treated samples, particularly those modified with NaOH and sodium ethoxide. For all modified samples there is a broad band at (3030–3500) cm^−1^ related to (N–H) stretching vibration, the band of –CN group at 2245 cm^−1^ somewhat decreased, and accompanied by the appearance of a small peak at 1650 cm^−1^ which is the distinctive band of the amide functional group^31^. The FTIR of NaOH-modified AFW discloses that the intensity of the bands of the carbonyl group at 1740 cm^−1^ and 1735 cm^−1^ were decreased relative to the unmodified AFW. While NaOH does not directly react with nitrile groups, it can indirectly influence them through the hydrolysis of adjacent ester groups. However, it does not directly react with nitrile groups. It may indirectly affect the nitrile group. The intensity of the nitrile group decreased after modification with Na-ethoxide. Na-ethoxide can hydrolyze the nitrile groups to form amide groups (–CONH_2_) and sodium carboxylate groups at 1632 cm^−1^ and 1570 cm^−1^ respectively depending on the reaction conditions^32^.

**Fig. 2:**
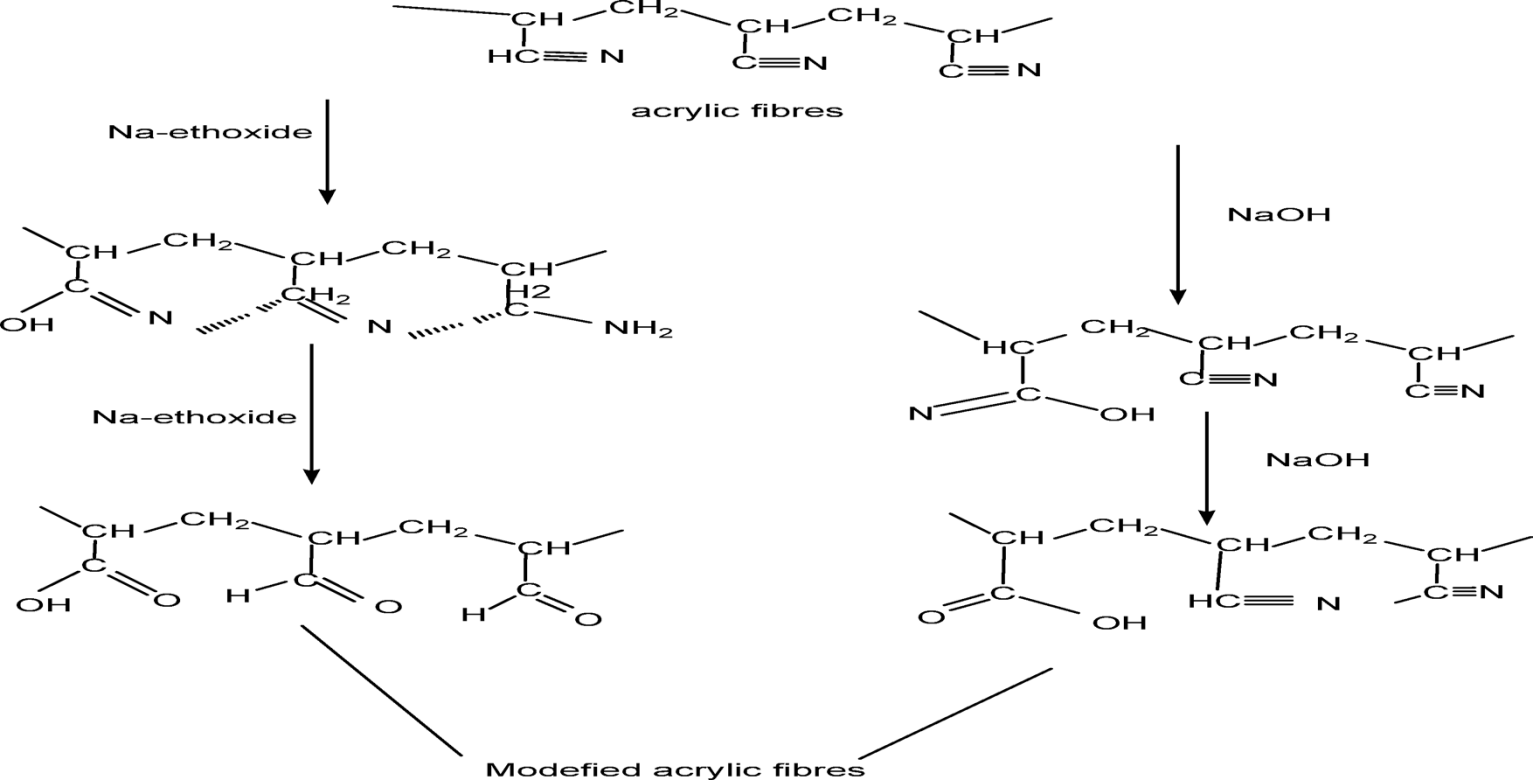
The chemical reactions of acrylic fibers with sodium ethoxide and sodium hydroxide.

Alkaline hydrolysis of acrylic fibres waste in alkaline media using either Na-ethoxide or NaOH affects the hydrolysis process in terms of resulting product formation and the mechanism involved^21^. Overall, the hydrolysis of nitrile groups in polyacrylonitrile polymer is affected when modified with 1 M NaOH at 95 °C for 1 h. The hydrolysis reaction was slower than that modified with 1 M Na- ethoxide at 95 °C for 1 h. This reaction leads to the formation of hydroxyl or amid group as shown in Fig. 2.

### Scanning electron microscope (SEM) of modified AFW

**Fig. 3:**
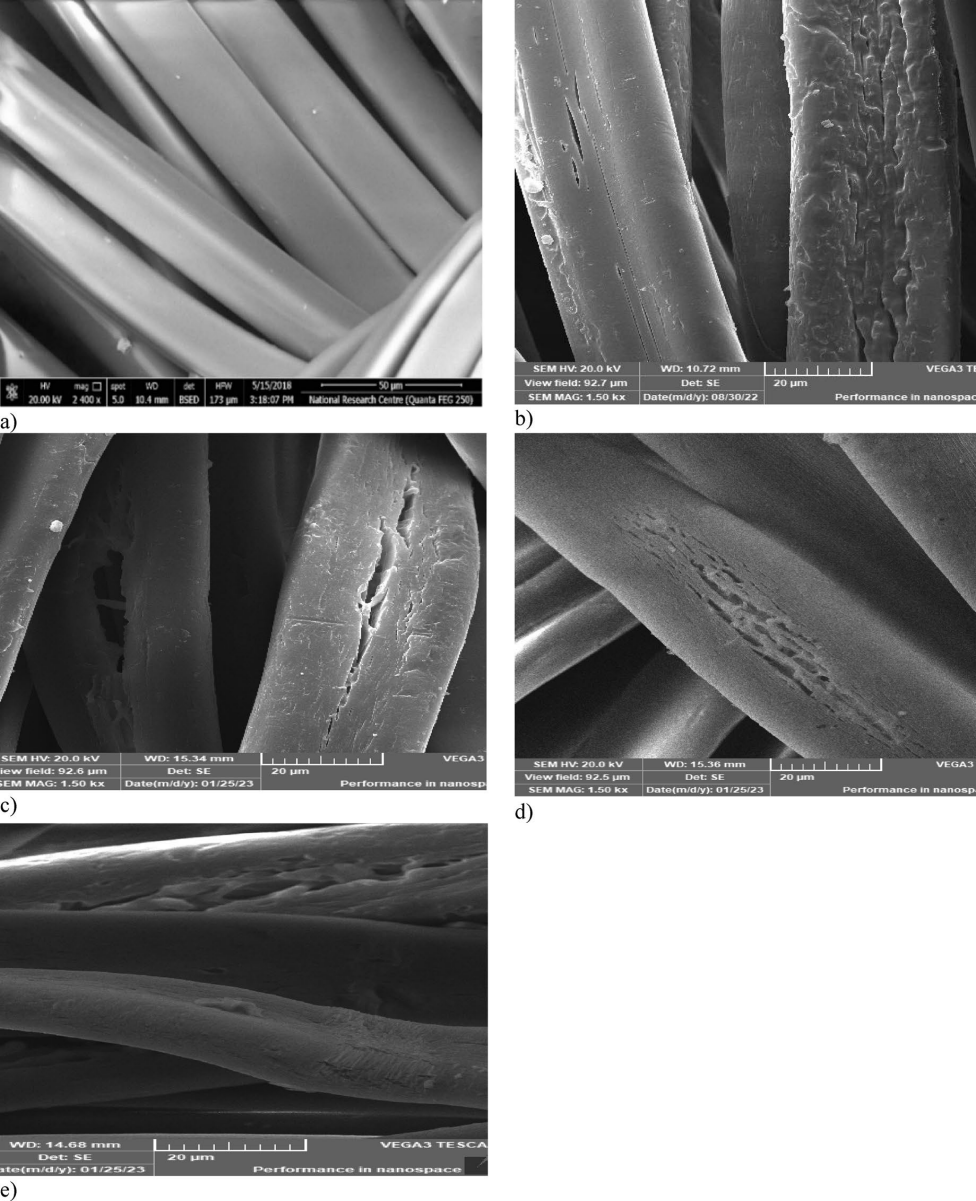
Scanning Electron Microscopy (SEM) images of unmodified and Modified ACFW. (**a**) Unmodified ACFW. (**b**) 1 M NaOH, 120 min, 95 °C, 1 h. (**c**) 1 M NaOH/DMF 30%V/V, 70 °C, 1 h. (**d**) 1 M NaOH/ethanol 50**/**50, 95 °C, 1 h. (**e**) 1 M Na-ethoxide, 95 °C, 1 h.

Figure 3a–e depict the surface morphology of unmodified and modified AFW observed under scanning electron microscopy. Figure 3a shows that unmodified AFW has a smooth surface and homogenous structure. Figure 3b–e display the surface morphology of hydrolyzed ACFW under different conditions. Hydrolysis not only altered the chemical structure of macromolecular fibers but also created porous structures because of the partial dissolution of the AFW surface. Additionally, the roughness is more noticeable for samples modified with NaOH (water/ethanol) or NaOH (water/DMF) and Na-ethoxide compared to those modified with pure NaOH. On the other hand, hydrolyzed AFW has clear signs of etching and produces more uneven defects and cracks, which may be the effect of the solvents^16^. Hydrolysis disrupts the compact structure of the acrylic fiber waste (AFW), potentially facilitating enhanced penetration of dye molecules into the fiber matrix. This structural alteration, particularly the increased fragmentation of polymer chains induced by hydrolysis as shown in Fig. 3e, may contribute to the elevated adsorption capacity observed in the Na-ethoxide-modified samples. The generation of increased pores spaces and amorphous regions within the hydrolyzed AFW provides more available sites for dye molecule adsorption.

### The thermogravimetric analysis (TGA) of modified AFW

**Fig. 4:**
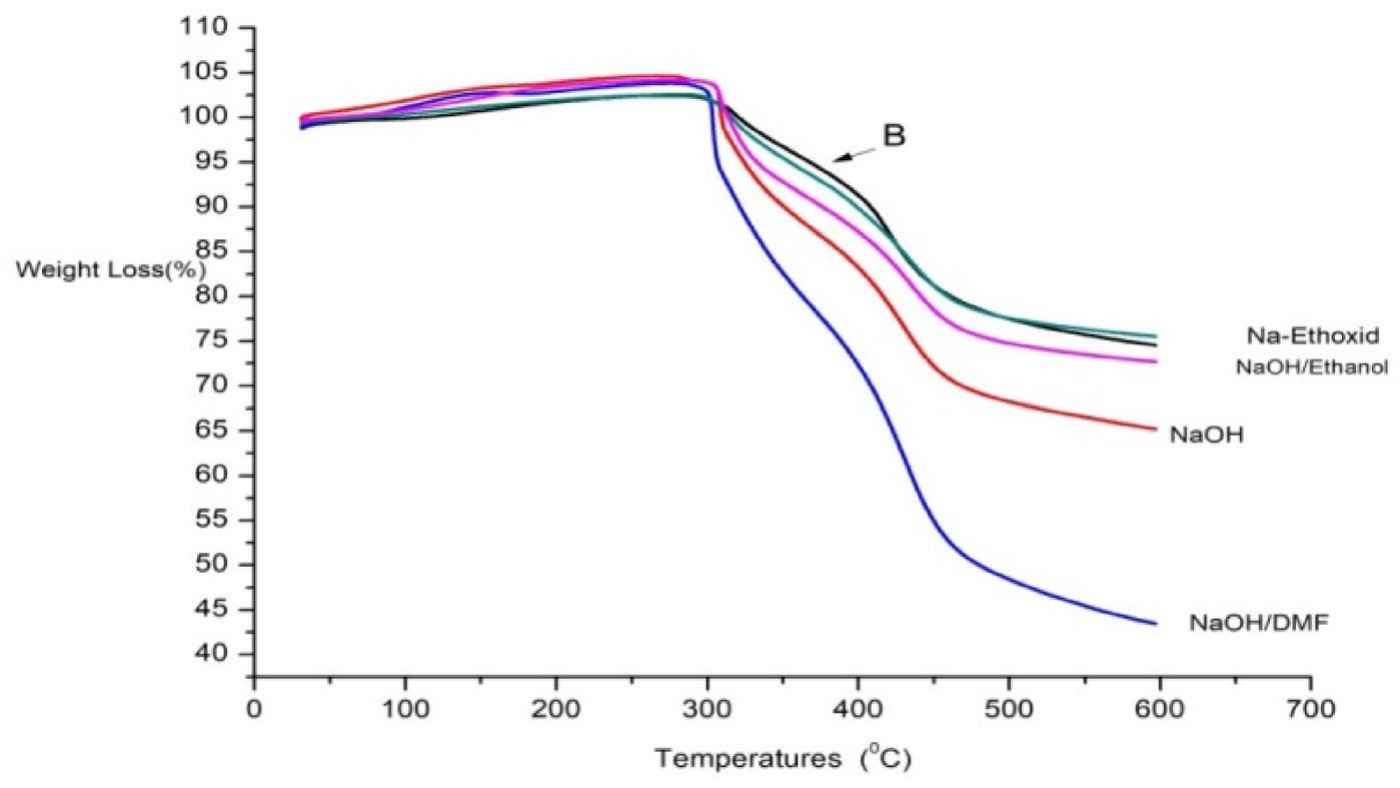
TGA diagram of unmodified and modified acrylic fibres waste.

The mathematical analysis of the thermogravimetric of the modified AFW (decomposition temperature, mass loss % and residual mass %) by TGA software were presented in Table 2. The loss in weight % of the modified acrylic fibres waste with NaOH is 17.2%, 25 and 32% at 310 °C, 390 °C and 480 °C respectively. On other hand, the modification of AFW with NaOH/ DMF has obtained high loss in weight %, due to effect of DMF. It is well known that DMF opening up the fibres structure, which led to more hydrolysis^33^. The observed mass loss percentages for acrylic fiber waste (AFW) modified with NaOH/ethanol were relatively elevated compared to the unmodified AFW, suggesting the influence of the hydrolysis process. However, this effect was less pronounced than that observed with NaOH/DMF modification. Mass loss ranged from 7.5% at 392 °C to 23.7% at 475 °C. Notably, the residual mass for Na-ethoxide-modified AFW was 48.4%, indicating a significant impact on thermal stability. The mass loss percentages for Na-ethoxide-modified AFW were 10.5% at 308 °C, 16.2% at 400 °C, and 24.9% at 455 °C, which are comparable to those of the unmodified AFW. The overall decrease in mass can be attributed to the thermal decomposition of higher molecular weight species and the transformation of crystalline regions within the polymer matrix into amorphous regions^34^. Figure 4 shows the TGA of untreated acrylic fabric and treated acrylic fiber waste. It was found that the untreated acrylic fabric indicates weight loss occurring in two steps: first, about 15% at temperatures of 223 °C and 279 °C, followed by further weight loss of approximately 25% at initial temperatures of 299 °C and 301 °C. This weight loss can be related to evaporation reactions, such as the evaporation of gases like NH3, CO2, and water vapor. In the case of the hydrolyzed sample, there was a loss in weight in the range of 291–441 °C, which may be related to the thermal decomposition of the monomers present in acrylic fibers (ester, sulfonic, hydroxyl, etc.) and the liberation of CO2 gas^21^.

**Table 2:** Thermogravimetric (TGA) data of unmodified acrylic fibre waste and modified acrylic fibre waste.

Sample	First stage		Second stage		Third stage		Residual weight %
	T_d_ (°C)	Weight loss%	T_d_ (°C)	Weight loss%	T_d_ (°C)	Weight loss%	
Unmodified AFW	307	10.1	401	17.1	450	25.3	47.5
NaOH	310	17.2	390	25	480	32	25.8
NaOH/DMF	300	13.5	380	26.4	460	52.3	7.8
NaOH/ethanol	309	7.5	392	18.5	475	24.7	49.3
Na-ethoxide	308	10.5	400	16.2	455	24.9	48.4

### Surface area measurement (BET) MAFW

N2 physisorption isotherms were analyzed according to the IUPAC classification^35^. This type is characteristic of non-porous or macroporous adsorbents. The isotherms exhibited hysteresis loops of Type H3 with no limited adsorption at high P/Po values, indicating the presence of non-rigid aggregates of plate-like particles that create slit-shaped pores. The results of unmodified and modified acrylic fibres waste, with 1M Na-ethoxide were analyzed.

The surface area measurement is supported by obvious SEM images showing modified acrylic fibres waste with Na-ethoxide. Table 3 shows the specific surface areas of both materials were determined using the BET equation applied. There was high difference in the specific surface areas (BET) of modified acrylic fibres waste from 1.0978 m^2^/g for unmodified to 1.4159 m^2^/g for modified acrylic fibres waste, with Na-ethoxide. This consistency aligns with the indication of a non-porous or macroporous nature of the material inferred from the N2 physisorption isotherm shape. In conclusion, the modified material is non-porous, and the surface area is attributed to inter-particle voids (macroporous)^36^.

**Table 3:** BET area, pore size, and volume for untreated and treated acrylic fibre waste various treatment.

Samples code	S_BET_ ((m^2^g^−1^))	Total pores volume (cm^3^g^−1^)	Pore width (nm)	Mean pore diameter (nm)
Unmodified acrylic fibres waste	1.0978	0.2522	0.4422	12.187
Untreated acrylic fibres waste with Na-ethoxide	1.4159	0.3253	6.2844	23.814

**Fig. 5:**
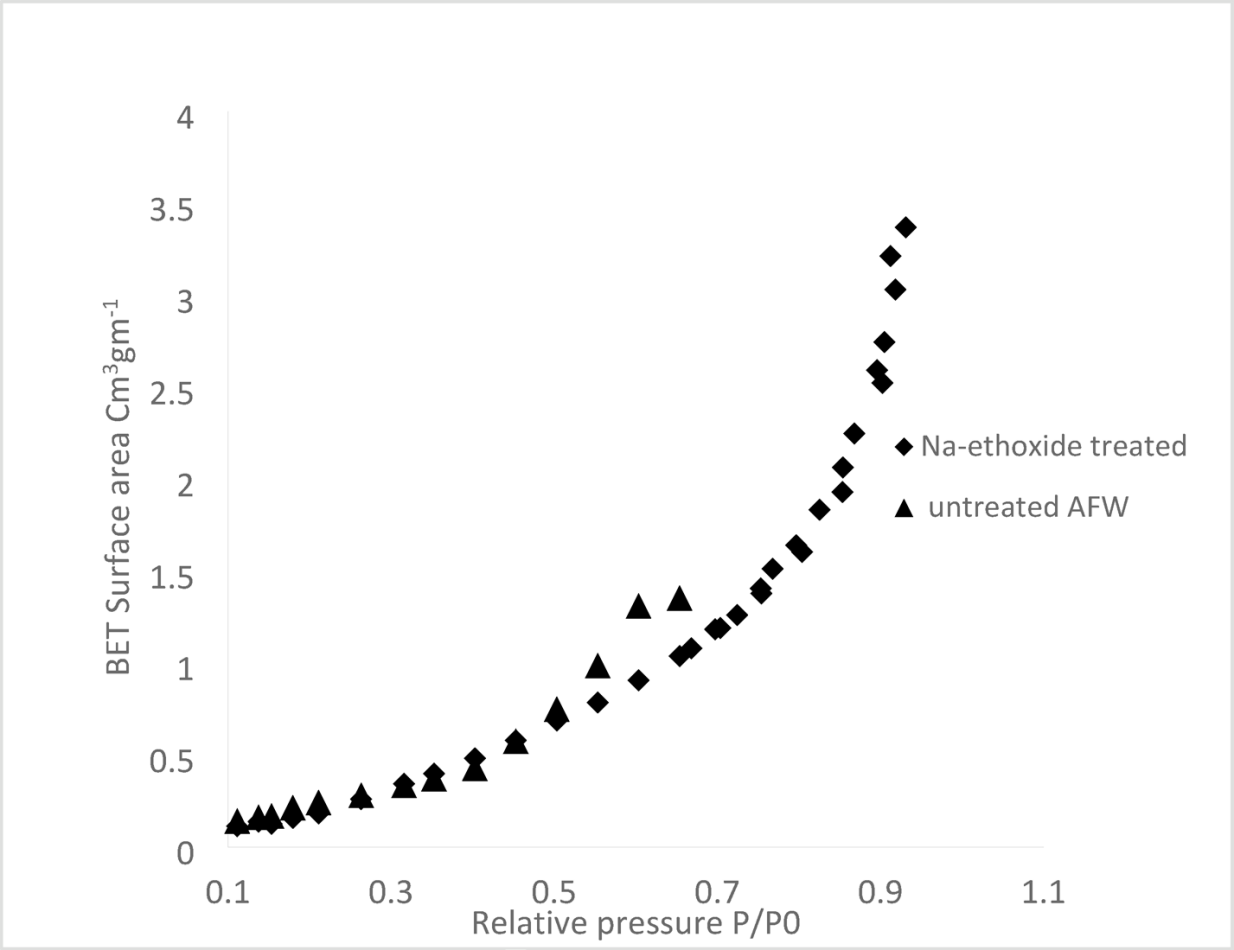
BET-adsorption isotherms of unmodified AFW and modified with Na-ethoxide.

Figure 5 displays the specific surface area BET of unmodified and modified AFW with Na-ethoxide. The modified AFW fibres exhibit a significantly increased surface area than the unmodified one. The abrupt loading transition in the P/P_o_ range of 0.1–1 suggests a uni-modal pore size distribution. The presence of micro and mesoporous confirms structures, with unrestricted monolayer adsorption at high P/Po values.

**Fig. 6:**
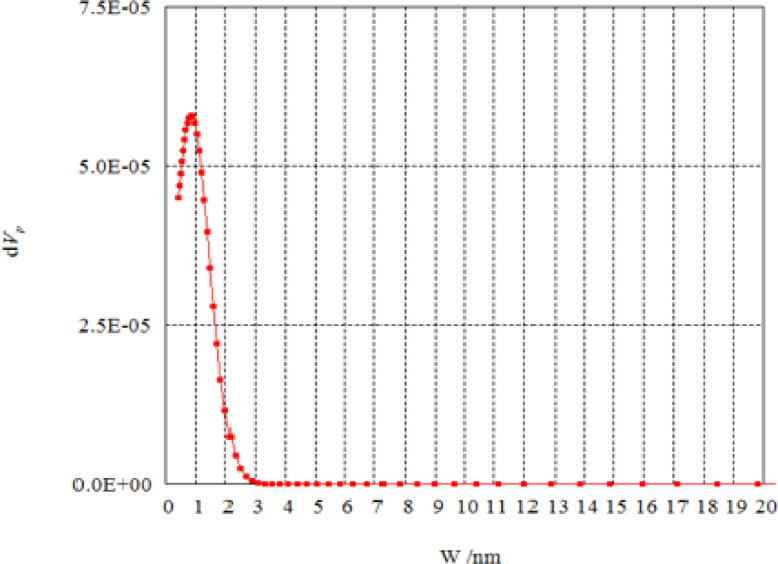
Pore size distribution of unmodified AFW.

**Fig. 7:**
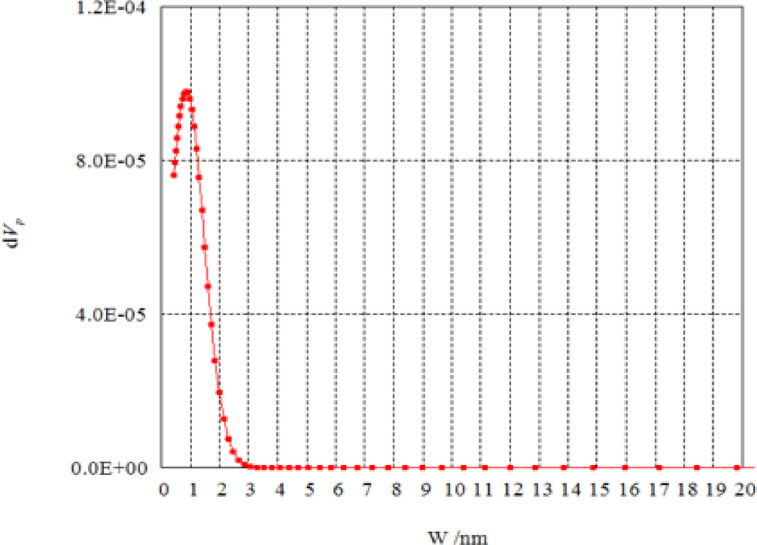
Pore size distribution modified AFW with Na-ethoxide.

Figures 6 and 7 show the pore size distribution of unmodified AFW and modified AFW with Na-ethoxide. Samples exhibit a sharp peak at a radius of 0.442 nm and 6.2844 nm indicating mesoporosity for unmodified AFW and modified AFW with 1M Na-ethoxide respectively. The pore size was increased from 12.187 nm for unmodified to be 23.814 nm for modified acrylic fibres waste. The effect of hydrolysis using Na-ethoxide creates some changes in the surface structure of the MAFW fibre. The increase in the surface area provides more active sites for interaction with dye molecules. This porosity helps in the adsorption of larger amounts of dye molecules, even in complex wastewater compositions^36^. Therefor, the MAFW included suitable regions for the adsorption of both MB and AR182 molecules.

### Removal of basic and acid dyes using acrylic fibres waste

#### Removal of basic and acid dyes using modified acrylic fibres waste with sodium hydroxide

##### Effect of NaOH concentrations

**Fig. 8:**
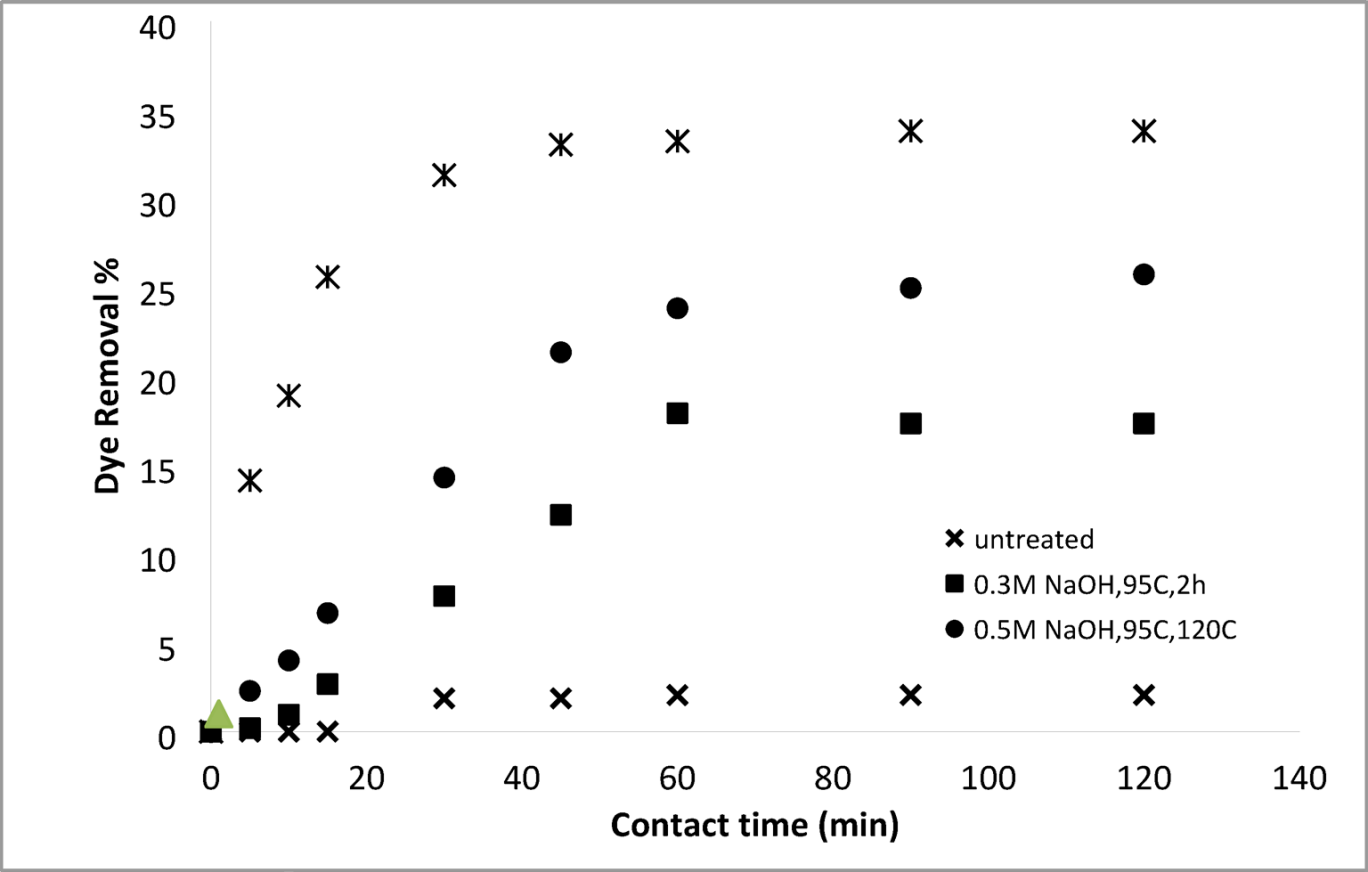
Dye removal efficiency of (AR 182) of modified AFW using different concentrations of sodium hydroxide. Condition of removal bath: AR182 (20 mg/L, (o.w.f), L: R 1:100, 1 h., 30 °C.

**Fig. 9:**
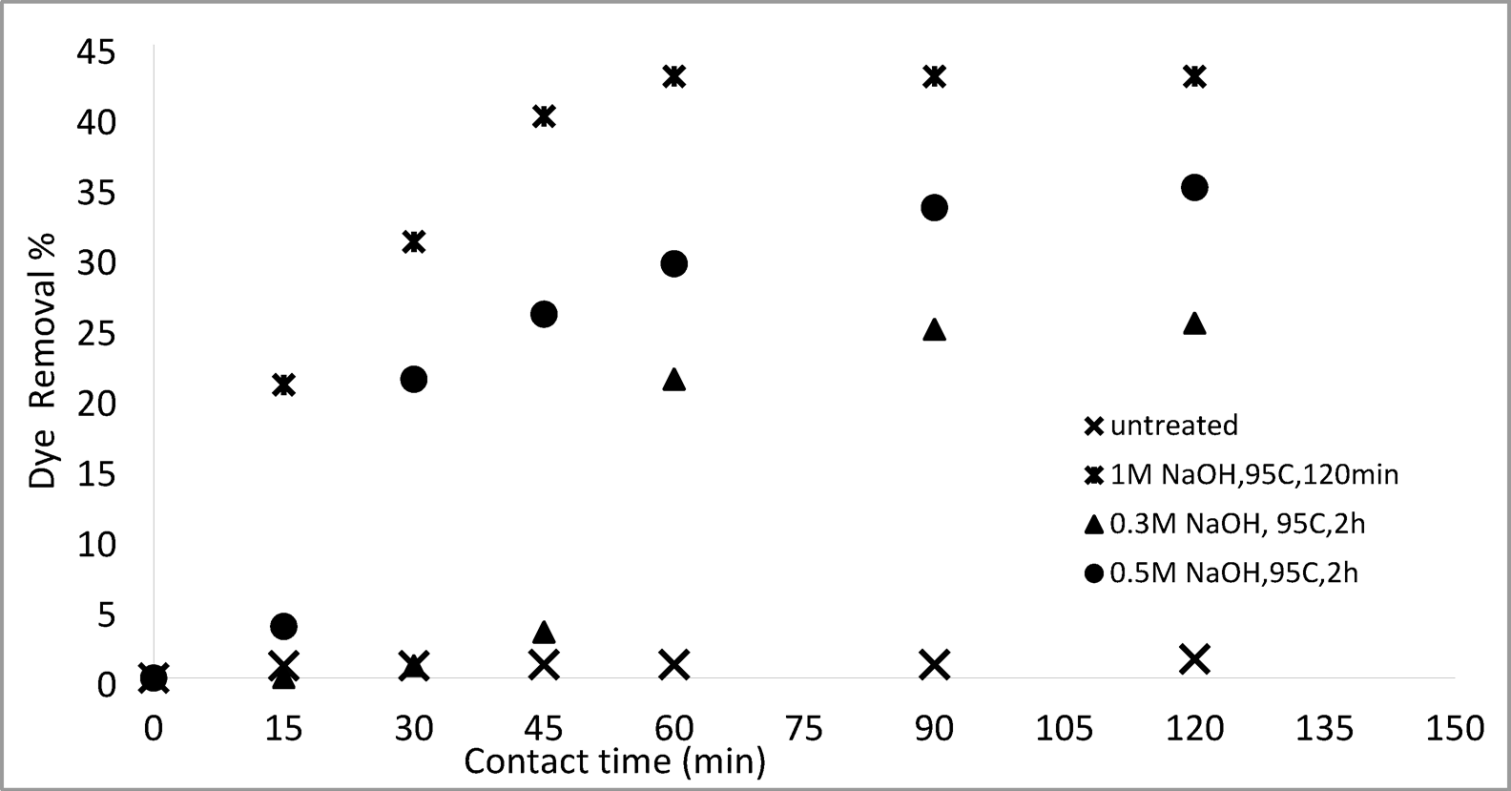
Dye removal efficiency of (MB) of modified AFW using different concentrations of sodium hydroxide. Condition of removal bath: MB182 (20 mg/L, (o.w.f), L: R 1:100, 1 h., 30⁰C.

The effect of NaOH concentration on the adsorption of AFW, as adsorbent material, was investigated. The concentration of dye removal bath was 20 mg/L with a pH 6.4 for MB and 4.8 for AR182. The temperature of the removal bath was 25 °C. Figures 8 and 9 depict the effect of NaOH concentration on the removal percentage of MB and AR182 dyes using modified AFW. However, after modification with 0.3 M NaOH, MB adsorption was enabled withe both the percentage and rate of removal increasing steadily NaOH concentration was raised to 1.0 M. This suggests that the functional groups created by NaOH play a crucial role in MB adsorption. The surface etching as shown in Fig. 3 and the formation of more voids as shown in Fig. 7 and amorphous regions may also contribute in the adsorption of MB. After 60 min of contact with the AFW modified by 1.0 M NaOH 42% of MB was removed. Additionally, the removal percentage increased gradually with increasing contact time. The high initially adsorption rate of MB is attributed to the availability of more adsorption sites on the surface of the adsorbent^37^.

On the other hand, the modification of AFW by NaOH affects the removal percentage of AR182 dye in a similar way to MB. Specifically, the removal of AR182 dye was negligible using the unmodified ACFW and increased significantly and gradually with the increasing concentration of NaOH. The removal of AR182 dye was 2.2% using the unmodified AFW and increased to 20%, 23.5%, and 33.5% using the AFW modified by 0.3 M, 0.5 M, and 1.0 M NaOH, respectively^38,39^. This adsorption process may be influenced by attractive Van der Waals forces and electrostatic attractions.

#### Removal of basic and acid dyes using modified acrylic fibres waste with sodium hydroxide mixtures (ethanol or DMF)

##### Effect of NaOH concentrations ethanol or DMF solutions

**Fig. 10:**
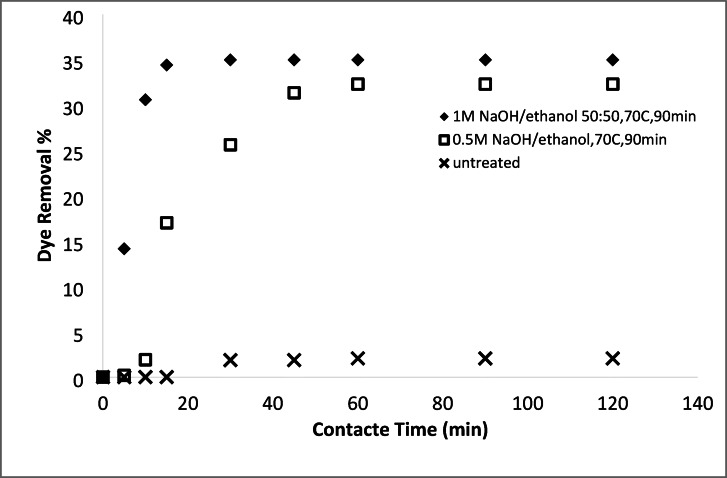
Dye removal efficiency of (AR182) of modified AFW using different concentrations of NaOH/ethanol. Condition of removal bath: AR182 (20 mg/L, (o.w.f), L: R 1:100, 1 h., 30 °C.

**Fig. 11:**
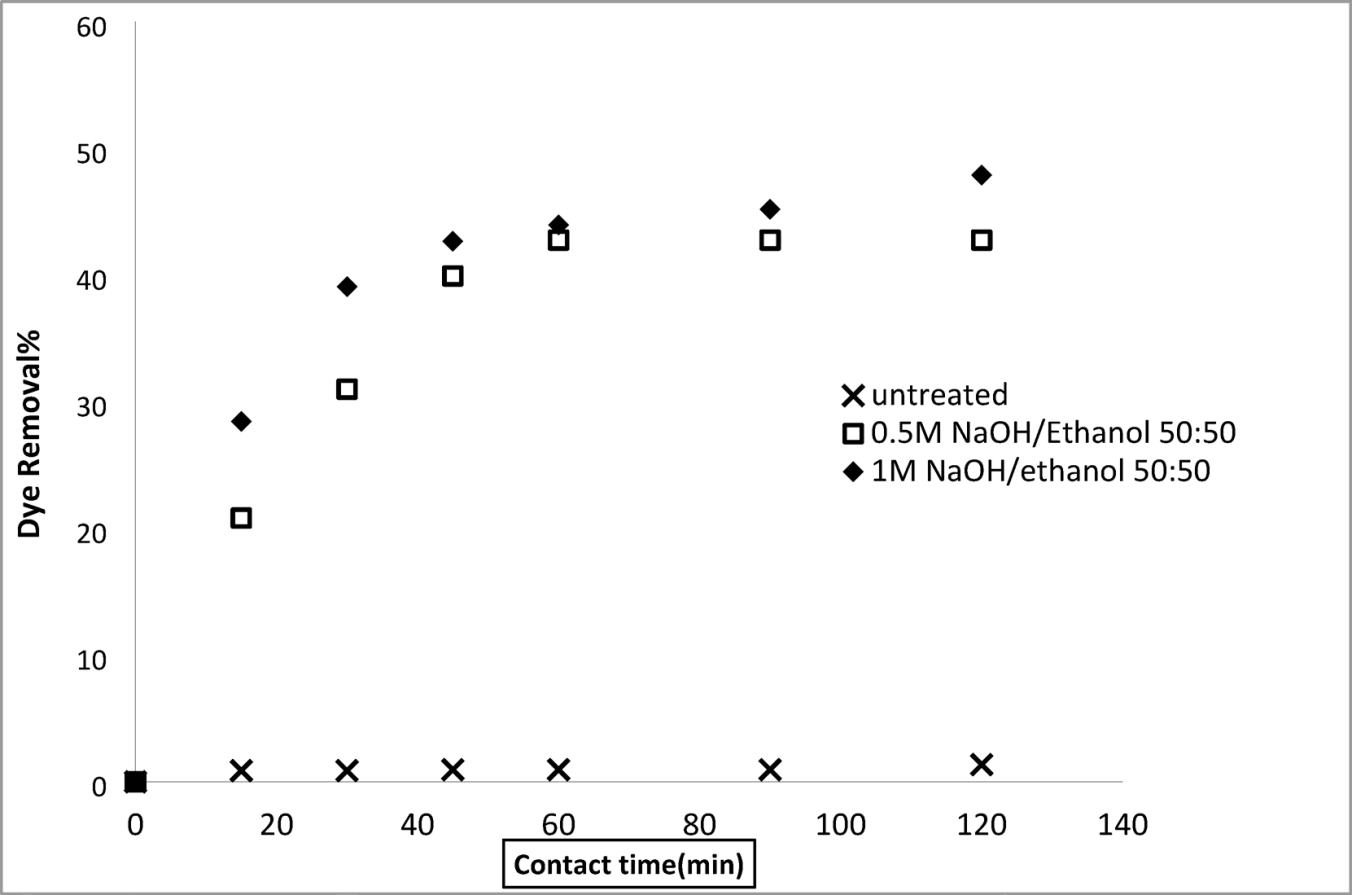
Dye removal efficiency of (MB) of modified AFW using different concentrations of NaOH/ethanol. Condition of removal bath: MB (20 mg/L, (o.w.f), L: R 1:100, 1 h., 30 °C.

**Fig. 12:**
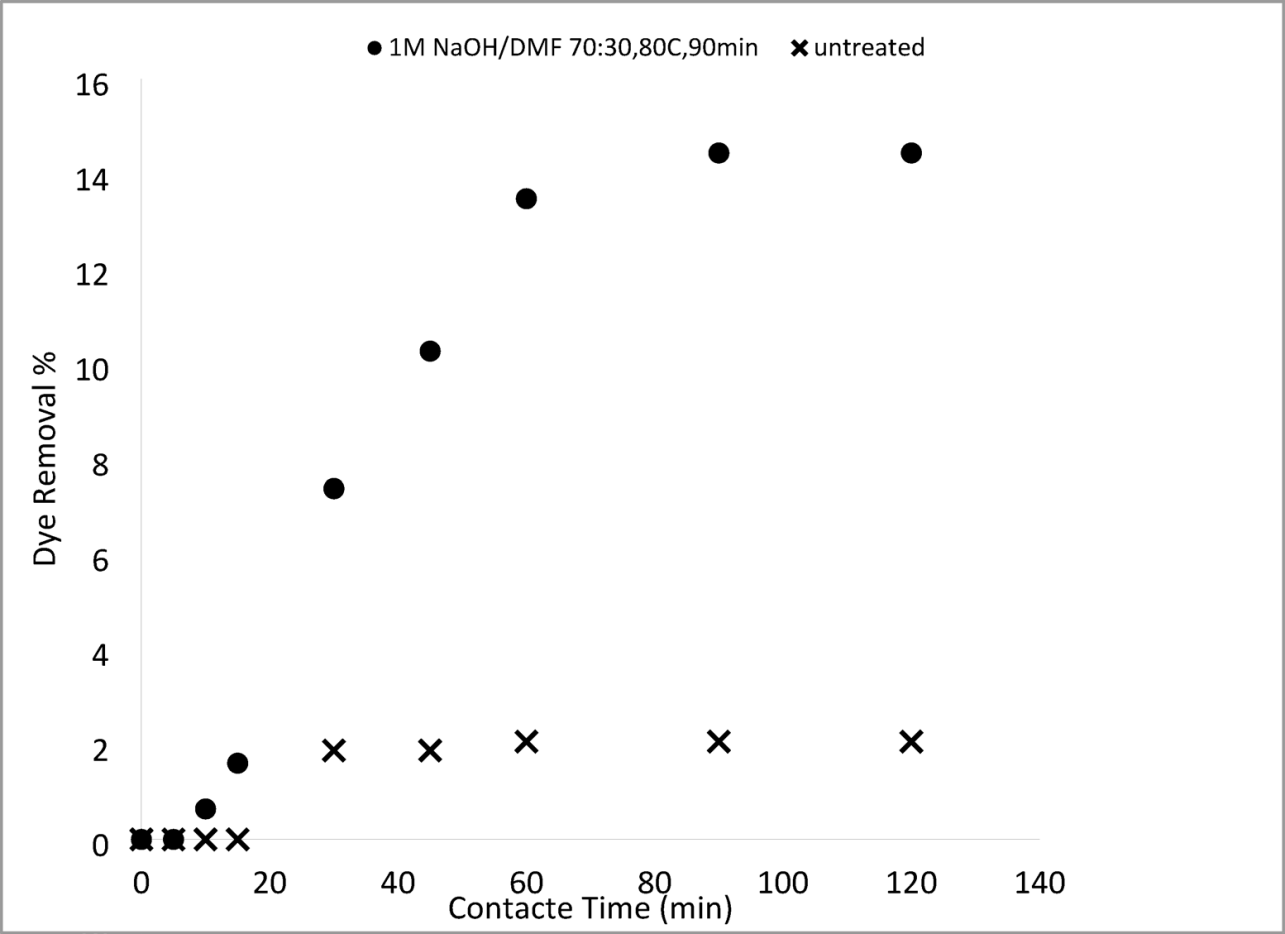
Dye removal efficiency of (AR128) by modified (AFW) with various concentrations of sodium hydroxide/DMF. Condition of removal bath: AR182 (20 mg/L, (o.w.f), L: R 1:100, 1 h., 30 °C.

**Fig. 13:**
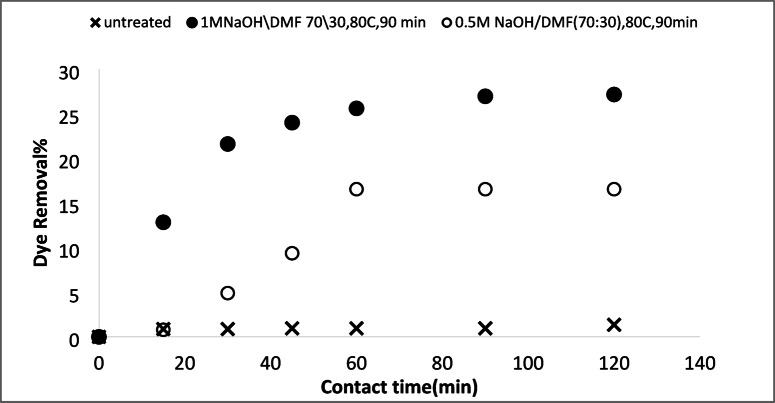
Dye removal efficiency of (M B) from modified (AFW) using various concentrations of sodium hydroxide/DMF. Condition of removal bath: MB (20 mg/L, (o.w.f), L: R 1:100, 1 h., 30 °C.

A series of solutions were prepared by mixing water with either ethanol or N, N-dimethylformamide (DMF) at varying ratios. Sodium hydroxide (NaOH) was then dissolved in these mixtures. These solutions were used to modify an AFW under different experimental conditions. The impact of NaOH concentration on the adsorbent’s performance in removing methylene blue (MB) and acid red 182 (AR182) was evaluated, following the experimental protocol outlined previously. Figures 10, 11, 12 and 13 demonstrate that the AFW modified with NaOH in either ethanol or DMF exhibited comparable adsorption capabilities, particularly at lower temperatures and shorter contact times. The removal percentages of MB and AR182 were found to increase with both NaOH concentration and the choice of solvent. The AFW modified with NaOH in solvent showed a higher adsorption capacity for MB than AR182, likely due to enhanced ionic interactions between newly formed functional groups on the adsorbent and the cationic MB dye. This suggests that the solvents facilitate the swelling of the adsorbent’s fiber structure, increasing the mobility of polymer chains, which enhances adsorption compared to modification with NaOH alone^39,40^.

#### Removal of basic and acid dyes using modified acrylic fibres waste with Na-ethoxide

##### Effect of Na-ethoxide concentrations

**Fig. 14:**
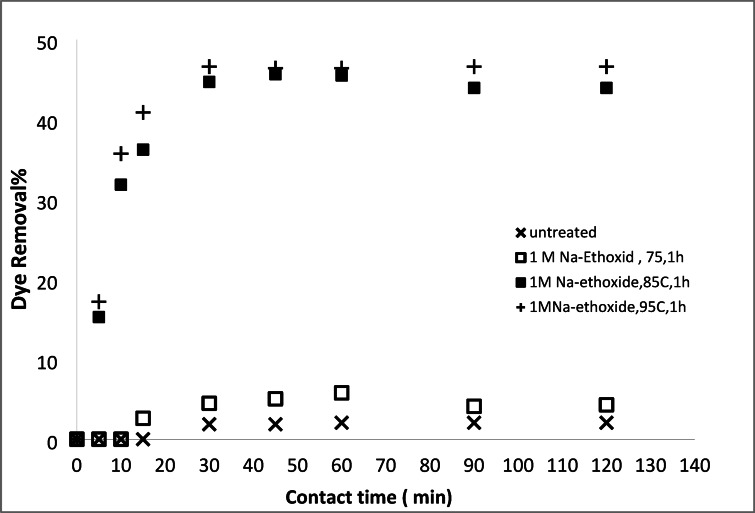
Dye removal efficiency of (AR182) of modified (AFW) using different concentrations of Na- ethoxide. Condition of removal bath: AR182 (20 mg/L, (o.w.f), L: R 1:100, 1 h., 30 °C.

**Fig. 15:**
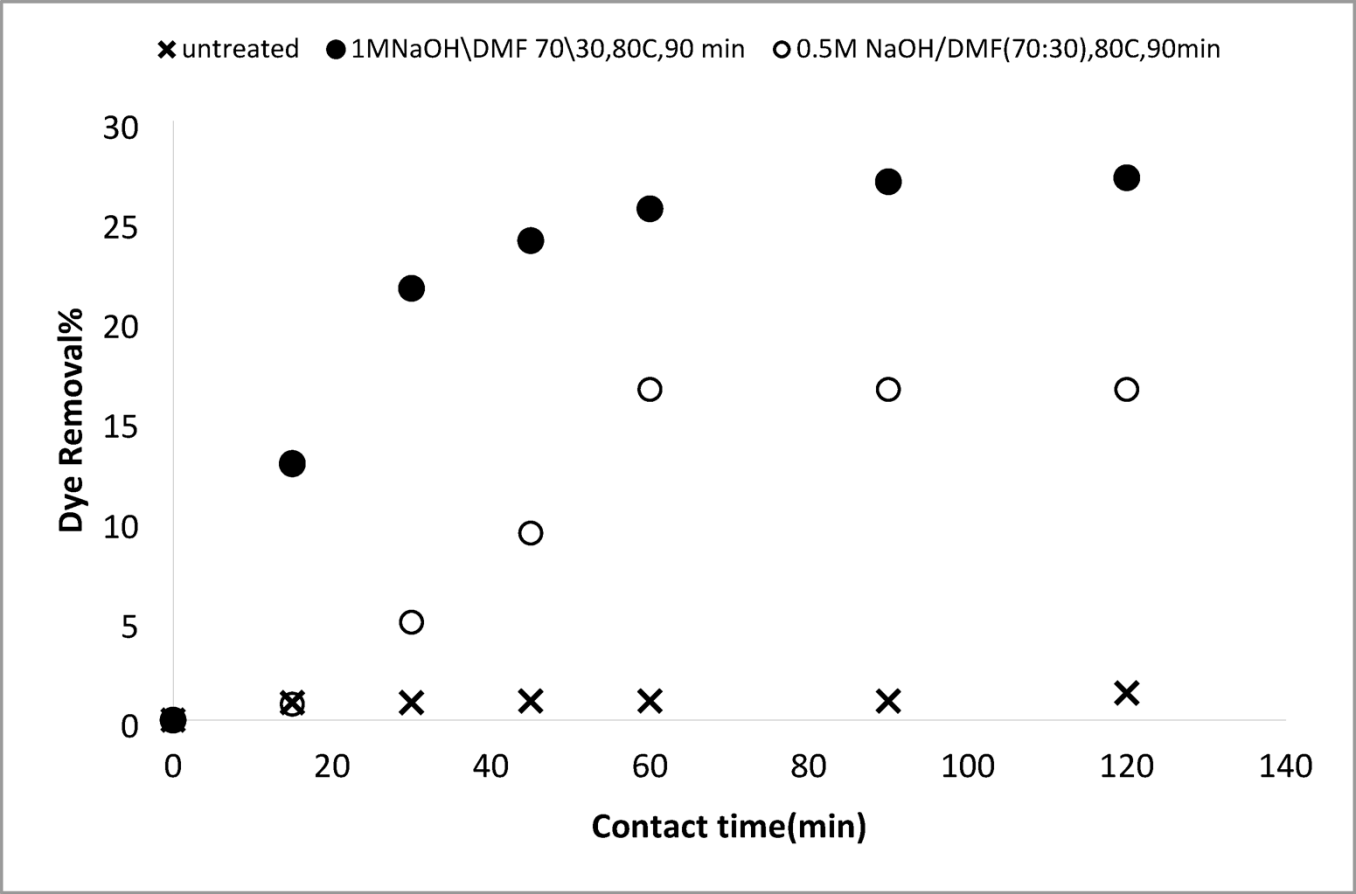
Dye removal efficiency of (MB) by modified (AFW) with various concentrations of sodium hydroxide/DMF. Condition of removal bath: MB (20 mg/L, (o.w.f), L: R 1:100, 1 h., 30 °C.

Figures 14 and 15 illustrate the removal percentages of AR182 and MB using AFW modified with sodium ethoxide at various temperatures, maintaining a constant concentration. The results indicate an increase in the removal percentage compared to unmodified AFW. Specifically, the removal percentage reached 44% for AR182 and 85% for MB. This improvement can be attributed to the strong alkaline hydrolysis, which leads to the formation of more active sites on the modified AFW surface, such as amide or sodium carboxylate groups. The enhanced removal of both AR182 and MB dyes on the modified fibers is likely due to the presence of these functional groups. The degree of dye adsorption is directly related to the extent of hydrolysis. Higher hydrolysis levels result in a greater removal percentage, particularly for MB. This is due to the strong ionic interaction between the newly formed functional groups and the cationic MB dye.

##### Optimization of the adsorption process of modified AFW

**Fig. 16:**
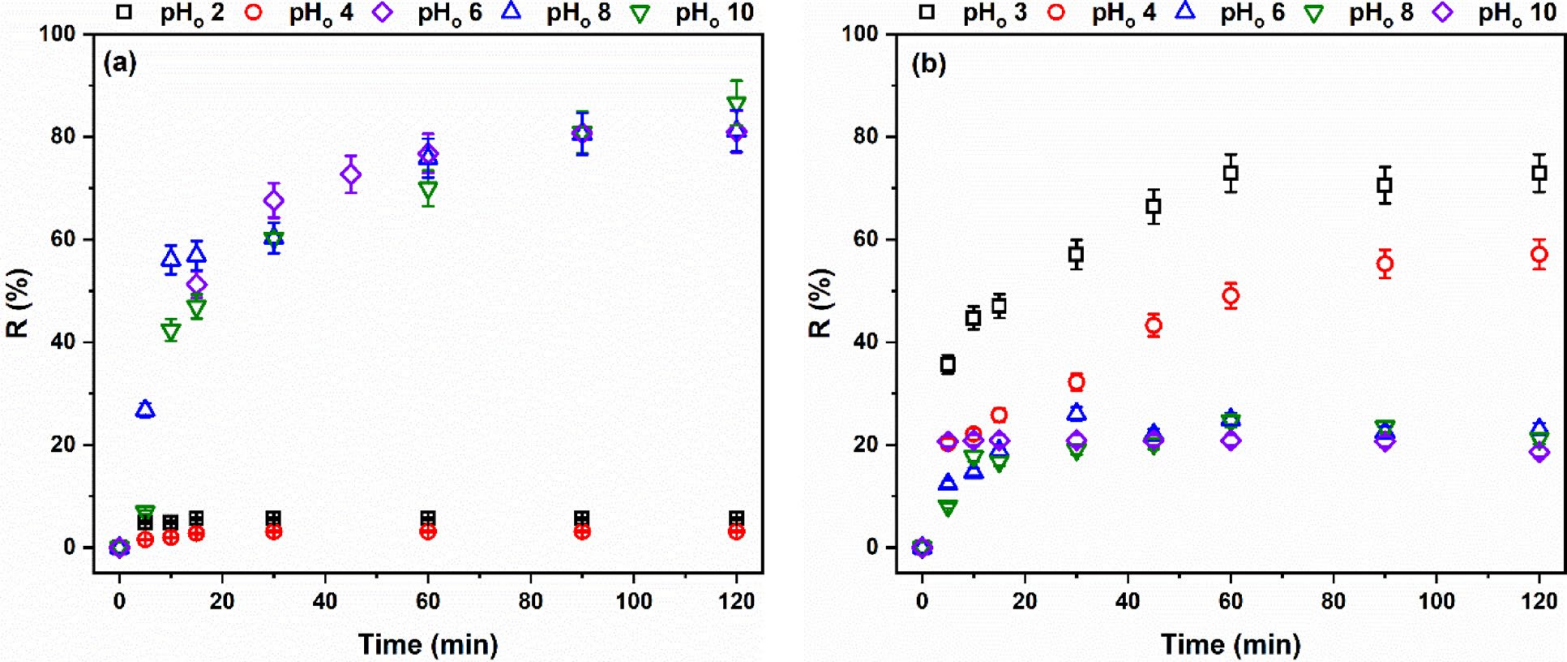
Effect of pH_o_ on the removal percentage of (**a**) MB and (**b**) AR182 of modified AFW. Removal condition: MB or AR182 (20 mg/L, (o.w.f), L: R 1:100, 1 h., 30 °C. Conditions of treatment: 1 M Na-ethoxide, 95 °C, 1 h.

Adsorption experiments were conducted across an initial pH range (pHo) of 2–10 to assess its impact on the adsorption process. Figure 16 reveals that MB adsorption was minimal at pHo 2 and 4, with removal percentages of 5.7% and 3.1%, respectively. However, a significant increase in adsorption was observed at pHo 6, reaching 81%. Further increases in pHo resulted in only marginal improvements, with removal percentages of 81% and 87% at pHo 8 and 10, respectively.

At pH_o_ 2 and 4 the functional groups of the modified ACFW become, protonated therefore repulsion with the cationic MB takes place. The competition between the hydronium ion and the cationic MB further decreased the removal percentage. At pH_o_ 6, 8 and 10 the functional groups of the modified AFW become deprotonated therefore attraction between the negatively charged and electron-rich functional groups and the cationic MB increases the removal percentage. Metwally et al., observed a similar effect of pH_o_ on the adsorption of MB by modified polyamide nanofibers^41^.

Conversely, the removal of AR182 exhibited an optimal performance at pHo 3, followed by a decrease up to pHo 6, after which it remained relatively constant. Specifically, removal percentages were 73% at pHo 3, 57% at pHo 4, 23% at pHo 6, 21% at pHo 8, and 19% at pHo 10. As previously mentioned, the functional groups of the modified AFW are protonated under acidic conditions. This protonation leads to electrostatic attraction with the anionic AR182 dye. As the solution’s acidity decreases, the degree of protonation diminishes, weakening the attractive forces and strengthening repulsive forces, resulting in a decline in removal efficiency. This observation aligns with numerous prior studies that have identified highly acidic pH levels as optimal for anionic dye removal^42,43^. The pH of the dye solutions likely plays a significant role in this differential behavior. Therefore, it seems that the different pH of the solutions of MB and AR182 explains the continuous increase in removal for MB and the attaining of the steady state for AR182.

**Fig. 17:**
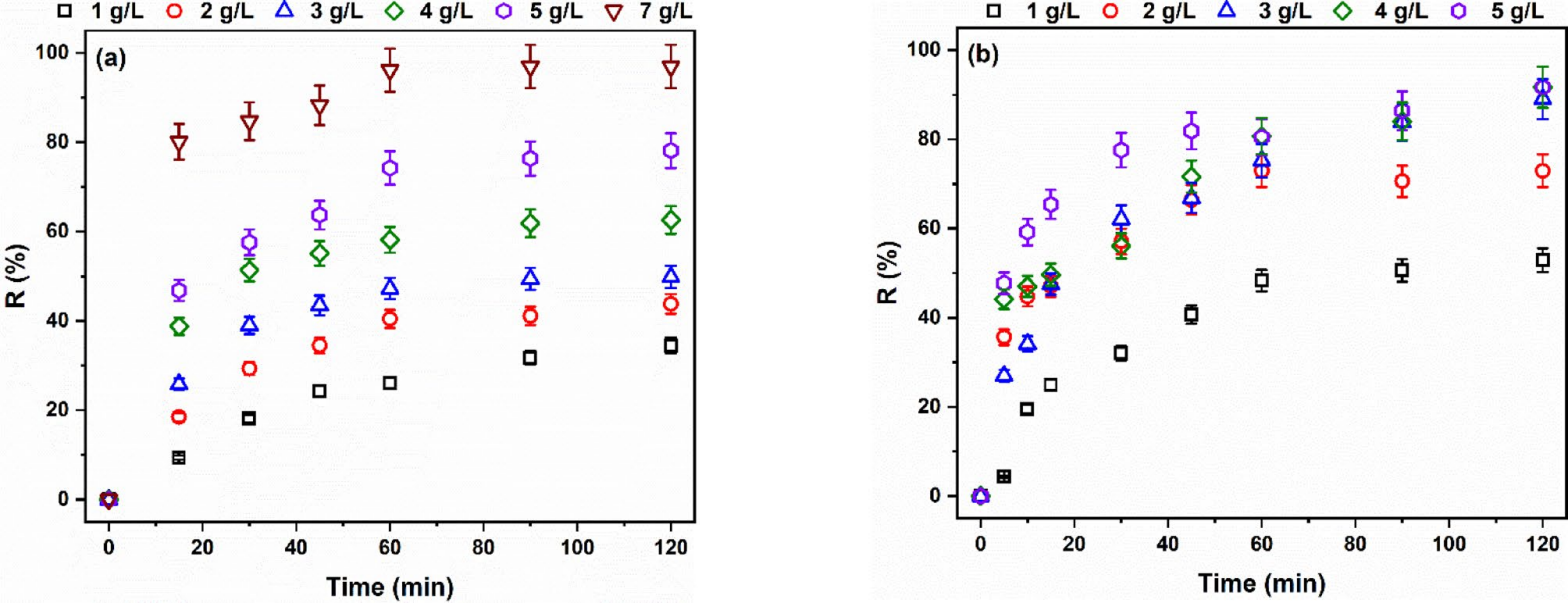
Effect of dosage on the removal percentage of (**a**) MB and (**b**) AR182 of modified AFW. Conditions of treatment: 1 M Na-ethoxide, 95 °C, 1 h. Condition of removal bath: Basic dye: pH 6.4, 1 h, 20 mg/L, (o.w.f), L: R 1:100, 30 °C. Condition of removal bath: Acid dye: pH4, 1 h, 20 mg/L. (o.w.f), L: R 1:100, 30 °C.

The removal percentage of MB and AR182 dyes were studied using different dosages of the modified AFW. The results are displayed in Fig. 17. MB removal exhibited a consistent increase with increasing AFW dosage. Specifically, removal percentages rose from 34% at 1 g/L to 97% at 7 g/L. On the other hand, the removal of AR182 showed a different trend. While initially increasing with dosage, it plateaued at higher concentrations. Removal percentages reached 53% at 1 g/L, 73% at 2 g/L, and 89% at 3 g/L, before marginally increasing to 92% at 4 g/L and 5 g/L. The continuous increase in MB removal can be attributed to the proportional increase in available adsorption sites with higher AFW dosages^44,45^. However, at higher dosages, adsorbent agglomeration can occur, reducing the effective surface area and accessible adsorption sites^46,47^. This explains the plateau observed in AR182 removal. Literature indicates that pH influences agglomeration. Consequently, the distinct pH values of the MB and AR182 solutions likely account for the continuous increase in MB removal versus the plateau observed for AR182.

**Fig. 18:**
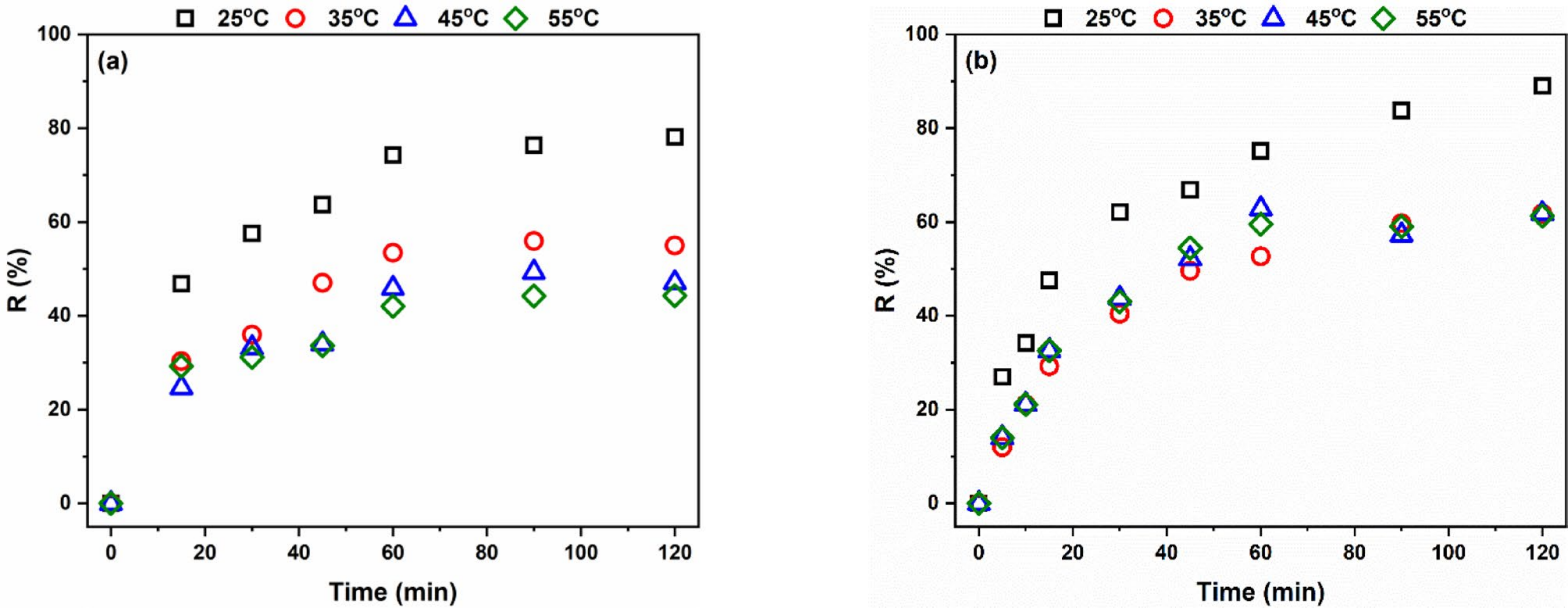
Effect of temperature on the removal percentage of (**a**) MB and (**b**) AR182 of modified AFW. Conditions of treatment: 1 M Na-ethoxide, 95 °C, 1 h. Condition of removal bath: Basic dye: pH 6.4, 1 h, 20 mg/L, (o.w.f), L: R 1:100, 30 °C. Condition of removal bath: Acid dye: pH4, 1 h, 20 mg/L. (o.w.f), L: R 1:100, 30 °C.

Temperature significantly influences the adsorption process. There are two possibilities, i.e., decreasing the removal percentage as the temperature increases, which may be attributed to the exothermic adsorption process, and increasing the removal percentage as the temperature increases, which may be attributed to the endothermic adsorption process^37^. Figure 18 shows that the removal percentage of both MB and AR182 decreased with increasing the temperature from 25 to 35 °C, and then insignificantly changed. The removal of MB was 78% at 25 °C and decreased to 55%, 47%, and 44% at 35 °C, 45 °C and 55 °C, respectively. Likewise, the removal of AR182 was 89% at 25 °C and decreased to 62%, 62%, and 61% at 35 °C, 45 °C and 55 °C, respectively. These results indicate that the adsorption of both MB and AR182 onto the modified AFW is an exothermic process^47^.

The duration of contact between the adsorptive and adsorbent has a significant impact on the removal percentage. To determine the equilibrium time for adsorption processes, the residual dye concentrations were measured at different times. The data reported in Fig. 18 indicates that the removal percentage of both dyes rose with time up to 60 min and then stayed almost steady. The presence of vacant adsorption sites and a high concentration gradient at the start of the contact between the dye and the adsorbent causes the observed high removal at the first 15 min. As time runs, the occupation of adsorption sites slows down the adsorption due to the limited number of vacant adsorption sites and the hindrance by the adsorbed dye molecules^48^. The removal of MB jumped to 47% in the first 15 min., steadily increased to 74% at 60 min., then marginally increased to 78% at 120 min. Similarly, the removal of AR182 after the first 15 min. was 46%, grew gradually to 72% after 60 min. and thereafter, no further appreciable adsorption occurred. These findings led to estimating 60 min. as the equilibrium time.

##### Adsorption kinetics of modified AFW

**Fig. 19:**
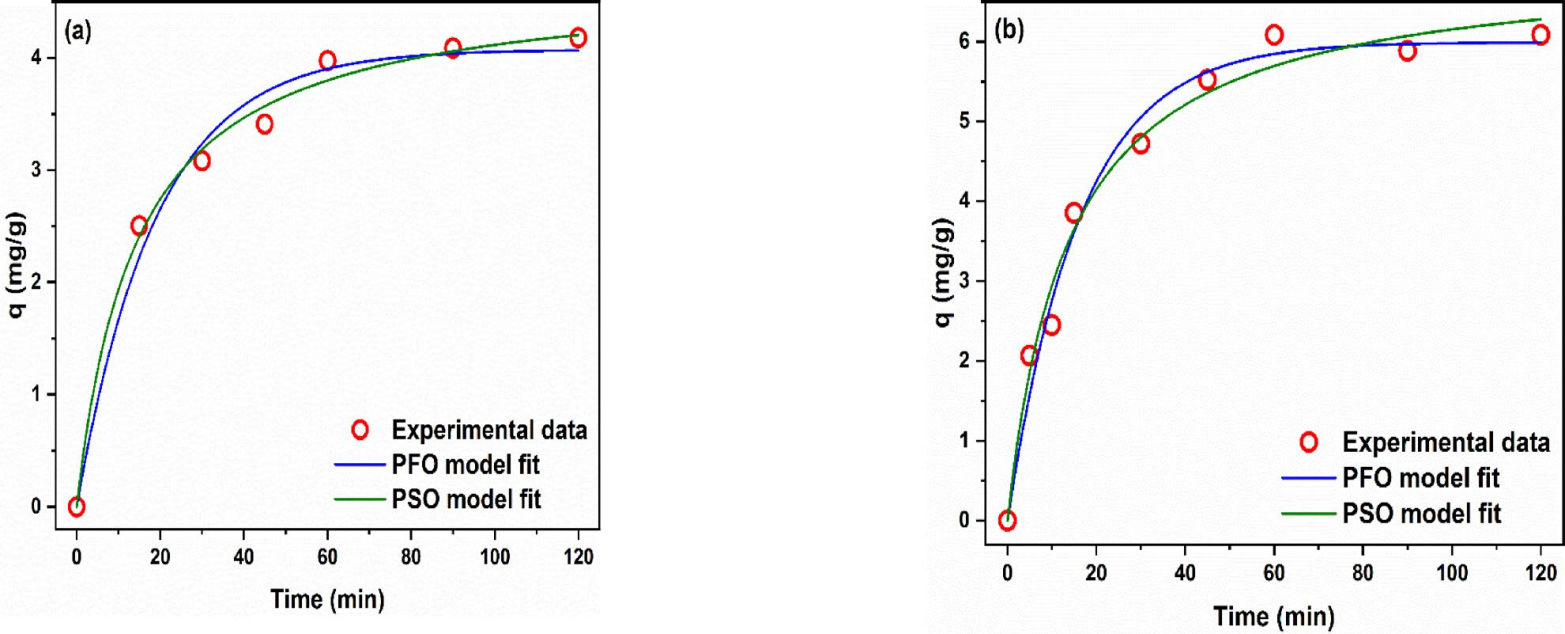
Adsorption kinetics of (**a**) MB and (**b**) AR182 of modified AFW. Conditions of treatment: 1 M Na-ethoxide, 95 °C, 1 h. Condition of removal bath: Basic dye: pH 6.4, 1 h, 20 mg/L, (o.w.f), L: R 1:100, 30 °C. Condition of removal bath: Acid dye: pH4, 1 h, 20 mg/L. (o.w.f), L: R 1:100, 30 °C.

The fit of the PFO and PSO models to the practical kinetic results was examined in order to realize the nature of the adsorption process. The Pseudo-First-Order (PFO) model assumes that the rate of occupation of adsorption sites is proportional to the number of unoccupied sites. This model is typically suitable for systems where physical adsorption is the predominant mechanism^25^. In the graph, the PFO model slightly underestimates the adsorption at early and late time points compared to the experimental data. The Pseudo-Second-Order (PSO) model assumes that adsorption follows second-order kinetics, often implying chemisorption involving valence forces through the sharing or exchange of electrons. This model appears to better fit the experimental data over the entire time range, particularly at equilibrium. Figure 19 and Table 4 show the fitting curves and parameters, respectively. According to Fig. 19 both PFO and PSO models represent the practical results well. In Fig. 19, with the PSO model being more accurate. The PSO model is an empirical reaction kinetics-based model that assumes a low initial concentration, abundance of adsorption sites, and irreversible adsorption^49,50^. The PSO model suites adsorption systems in which the adsorption is dominated by a chemical process A similar observation has been frequently reported before and explained by the participation of several forces in the adsorption process^24,25^. However, according to the values of R^2^ and χ^2^ in Table 4 the PSO model represents the practical results more accurately than the PFO model because the R^2^ of the PSO model is higher than that of the PFO model and the χ^2^ is lower. The closeness of q_e_ derived from the PSO model (4.70 mg/g) and 6 mg/g for MB and AR 128 respectively to the obtained practical value (4.18 mg/g MB) and (6.9 mg/g AR128) confirms that PSO model provides a better overall fit than the PFO model, suggesting that chemical interactions (e.g., electron sharing or exchange) are more significant in MB and AR adsorption^51^. Accordingly, the fit of the experimental data to the PSO suggests the modified AFW has a large number of adsorption sites, a preponderance of chemical interactions between MB and the modified AFW, and irreversibility of the adsorption process. This result suggests that the adsorption of MB onto the modified AFW may be dominated by chemical process^50^.

**Table 4:** Kinetic models parameters for MB and AR182.

	MB		AR182	
	PFO	PSO	PFO	PSO
R^2^	0.984	0.994	0.985	0.984
χ^2^	0.041	0.016	0.082	0.084
K	0.053 ± 0.007	0.015 ± 0.002	0.062 ± 0.006	0.010 ± 0.002
q_e,model_	4.073 ± 0.131	4.703 ± 0.147	5.993 ± 0.165	6.997 ± 0.277

##### Adsorption isotherms of modified AFW

**Fig. 20:**
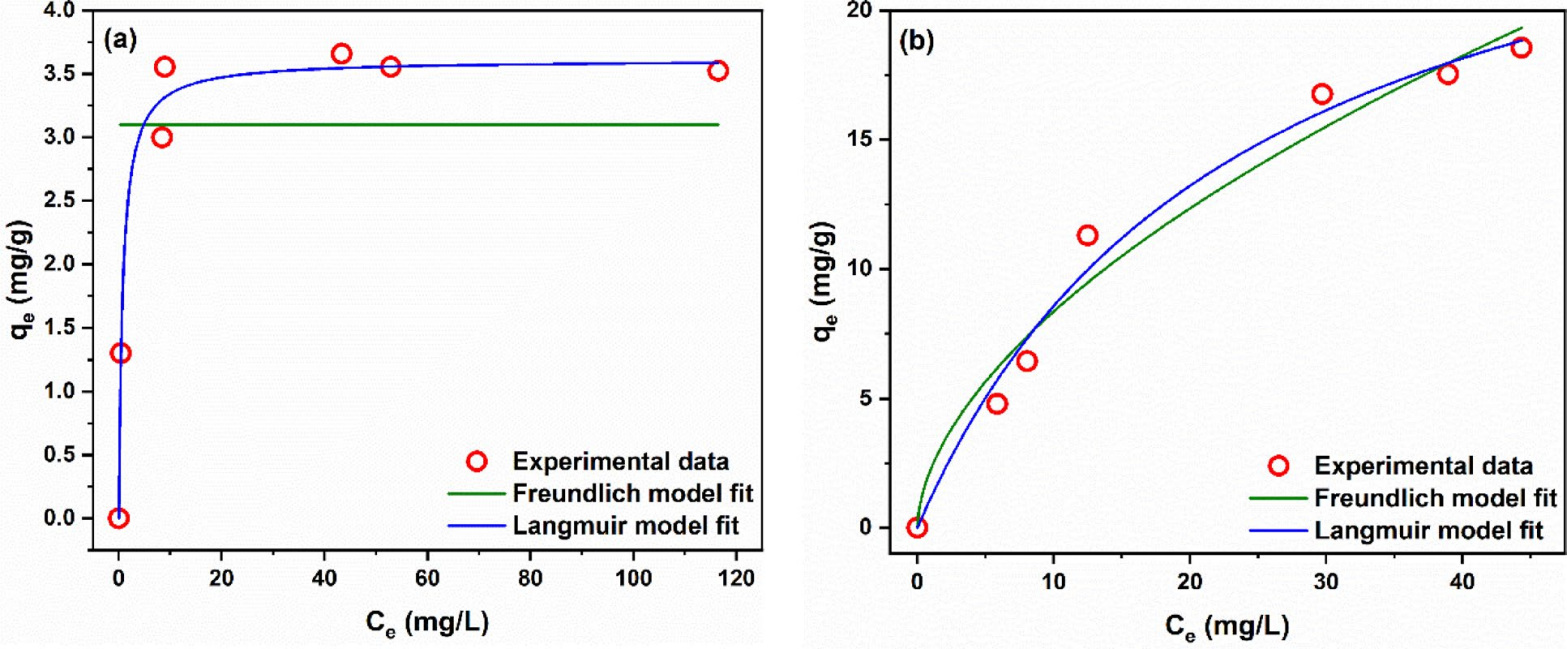
Adsorption isotherm of (**a**) MB and (**b**) of modified AFW. Conditions of treatment: 1 M Na-ethoxide, 95 °C, 1 h. Condition of removal bath: Basic dye: pH 6.4, 1 h, 20 mg/L, (o.w.f), L: R 1:100, 30 °C. Condition of removal bath: Acid dye: pH4, 1 h, 20 mg/L. (o.w.f), L: R 1:100, 30 °C.

The practical adsorption isotherms were analyzed by the Langmuir and Freundlich models. The plots of the resulting fitting curves are displayed in Fig. 20 and the values of the models’ parameters are shown in Table 5. Figure 18a clearly indicates that the Freundlich model failed to represent the practical isotherm of MB adsorption onto the modified AFW. The values of R^2^ and χ^2^ in Table 3 further confirm the unsuitability of the Freundlich model to the practical data of MB adsorption onto the modified AFW. On the other hand, Fig. 20b shows that the fitting curves of both Langmuir and Freundlich models match the practical data of AR182 well. However, the values of R^2^ and χ^2^ in Table 5 indicate the higher accuracy of the Langmuir model in representing the practical results than the Freundlich model. Thus, the Langmuir model can represent the adsorption isotherm of both MB and AR182 well. Langmuir model is a chemical adsorption model designed for adsorbents that have homogeneous adsorption sites where the adsorbate forms only one layer on the surface of the adsorbent and does not interact with each other^41,52^.

**Table 5:** Isotherm models parameters for MB and AR182.

	MB			AR182			
	Freundlich		Langmuir		Freundlich		Langmuir
R^2^	0.665	R^2^	0.987	R^2^	0.972	R^2^	0.987
χ^2^	0.829	χ^2^	0.033	χ^2^	1.748	χ^2^	0.829
K_F_	3.099 ± 0.372	K_L_	1.241 ± 0.277	K_F_	2.279 ± 0.564	K_L_	0.042 ± 0.009
n_F_	− 1.07849 × 10^21^ ± 0	q_m_	3.611 ± 0.095	n_F_	1.774 ± 0.227	q_m_	28.967 ± 2.833

The separation factor (R_L_) is a key character of the Langmuir model which indicates the nature and favorability of the adsorption process. An R_L_ value of zero implies irreversible adsorption, one implies linear adsorption, greater than one implies unfavorable adsorption, and between zero and one implies favorable adsorption. The value of R_L_ was calculated according to Eq. 12.

12 $${\text{R}}_{{\text{L}}} { = }\frac{{1}}{{{\text{1 + K}}_{{\text{L}}} {\text{ C}}_{{\text{o}}} }}$$

The calculated values of R_L_ for MB were 0.08–0.01 while for AR182 were 0.64–0.19. Since these values range between zero and one, the adsorption of both MB and AR182 onto modified AFW is favorable.

The theoretical monolayer saturation capacity of Langmuir (q_m_) is usually used to compare the performance of different adsorbent materials toward a specific pollutant. Table 5 compares the Langmuir maximum monolayer adsorption (q_m_) for MB and AR182 dyes adsorption onto various materials as well as modified AFW as adsorbent material^50–54^.

It can be seen 36.1 and 28.9 for MB and AR182 respectively onto modified AFW and has considerably higher qmax than the reported values for d-Zeolite, Freeze–dried agarose gee and Beech wood sawdust for basic dyes as well as fish schels for acid dyes as shown in Table 6. On the other hand the greatest value for other various adsorbent materials not deal with textile as waste which characterized are low cost. In the current work, the adsorption nature of both MB and AR182 by modified AFW exhibited by the Langmuir isotherm model. This can be attributed to the high R2 that was noticed in both states. These results were due to the homogenous nature of the adsorbent surface. The dyes adsorbed completely to the modified AFW surface due to presence of “active sites” until no further adsorption can be noticed and no noticeable further interaction between the adsorbed dyes molecules and the neighboring active sites^12,58–65^.

**Table 6:** Adsorption performance of different adsorbents for methylene blue and acid dye.

Adsorbent material	Adsorption capacity Q_m_ mg/g	References
MAFW*	36.81 (MB)	Current study
MAFW*	28.97 (AR182)	Current study
Fish Scales	145.3 (Acid blue 113)	^55^
Fish Scales	1.8, 2.7 and 3.4 (Acid Red 1, Acid blue 45, Acid Yellow 127 respectively)	^56^
Banana peel	18.2(AR)	^63^
CB-CTAB	19.15(AR)	^65^
Acrylic fibres membrane	57.1(MB)	^12^
activated carbon	249.1(MB)	^42^
d-Zeolite	10.82(MB)	^47^
NaClO2 treated KF	110.3(MB)	^48^
pumpkin seed hull	141.92(MB)	^24^
Freeze–dried agarose gee	10.4(MB)	^49^
PCMC/AA/acrylamide/GO	133.3(MB)	^50^
SCellulose/activated carbon	103.6(MB)	^52^
Carboxymethylcellulose/PAA GO	138.4(MB)	^53^
Sugarcane bagasse	136.99(MB)	^62^
Apricot stone	46.031(MB)	^63^
Beech wood sawdust	5(MB)	^64^

MAFW*(modified AFW).

##### Thermodynamic characterization of modified AFW

The viability and spontaneity of an adsorption process are calculated by thermodynamic parameters^65^. They are also important for the evaluation of adsorbents (i.e. physisorption, ion exchange or chemisorption). The thermodynamics studies play an important role in thoroughly understanding the adsorption process of both MB and AR182 dyestuffs in aqueous solutions. Thermodynamic adsorption is essential in considering the types and mechanisms of the adsorption process under variations of the solution temperature. Table 7 summaries the calculating performance of the parameters of thermodynamics for the adsorption process. A modified AFW with 1M Na-ethoxide solution was selected and the thermodynamic Eqs. 10 and 11 were applied to it.

**Table 7:** Determination of the thermodynamic parameters.

T (K)	ΔG° (kJ/mol)	ΔH° (kJ/mol)	ΔS° (kJ/mol·K)	ΔG° (kJ/mol)	ΔH° (kJ/mol)	ΔS° (kJ/mol·K)
	MB			AR		
298.15	− 3.15	− 42.58	− 0.13	− 3.15	− 19.46	− 0.06
308.15	− 1.84			− 1.23		
318.15	0.07			− 1.27		
328.15	0.63			− 1.25		

Temp. 25 °C, pH 2.8, Con 20 mg/L, dosage 3gm/L.

Conditions of treatment: 1 M Na-ethoxide, 95⁰C, 1 h.

For MB dye, the negative values of ΔG° at 25 °C and 35 °C indicate that the adsorption process is spontaneous at these temperatures. However, at 45 °C and 55 °C, ΔG° becomes positive, suggesting that the process is non-spontaneous under these conditions. The negative ΔH° and ΔS° values further indicate that the adsorption of MB is exothermic and accompanied by a decrease in randomness at the solid–solution interface.

In contrast, for AR182 dye, the low R^2^ value (0.59) obtained from the van’t Hoff plot suggests poor reliability of the calculated ΔH° and ΔS° values. Therefore, further interpretation of the thermodynamic behavior based on these parameters is not appropriate. This finding is consistent with Basirun et al., who also reported a low R^2^ value for the van’t Hoff equation in the adsorption of methyl orange onto chitosan-intercalated montmorillonite^66^.

##### Reusability study of modified AFW

Table 8 shows a study on the reusability of modified acrylic fiber waste as an adsorbent for MB. The modified acrylic fibers waste outperformed unmodified fibers in durability and efficiency. The Na-ethoxide modified fiber waste achieved 88% MB removal in the first cycle and maintained a removal efficiency of 47% after six cycles. In comparison, unmodified fiber waste only achieved 4.4% removal after six cycles, demonstrating the effective reusability of modified acrylic fiber waste for dye removal.

**Table 8:** Durability of adsorbent material for unmodified and modified AFW.

Number of uses	Removal efficiency % of MB	
	Unmodified	AFW with Na-ethoxide
1 time	10.54388	88.03333
2 time	10.54388	72.97309
3 time	4.961727	64.39176
4 time	4.961727	52.12045
5 time	4.708578	47.40071
6 time	4.494045	47.40071

Conditions of treatment: 1 M Na-ethoxide, 95 °C, 1 h.

## Conclusions

This study successfully converted acrylic fiber waste (AFW) into an effective adsorbent for removing both cationic (MB) and anionic (AR182) dyes from textile wastewater under ambient conditions (25°C). Alkaline hydrolysis using NaOH and Na-ethoxide proved to be highly effective in modifying AFW, introducing new functional groups and structural changes that significantly enhanced dye removal capabilities.

The study highlights the potential of Na-ethoxide as an efficient hydrolysis agent and emphasizes the importance of optimizing reaction conditions for maximum adsorption performance of the modified AFW. The findings contribute to the development of sustainable methods for removing dyes from textile wastewater, enabling adsorbent reuse and minimizing environmental impact. This approach offers both environmental and economic benefits.

For MB dye adsorption, the Freundlich isotherm model provided the best fit to the experimental data, indicating heterogeneous adsorption onto the adsorbent surface. The Freundlich isotherm model provided the best fit to the experimental data, as evidenced by its higher correlation coefficient (R^2^) compared to other models. Adsorption kinetics studies revealed that a pseudo-second-order kinetic model best described the experimental data.

FTIR analysis confirmed the presence of new functional groups on the modified AFW, which are responsible for the enhanced adsorption capacity. (It would be beneficial here to add a brief summary of the new functional groups that were detected by FTIR.

This novel approach utilizes waste acrylic fibers (AFW) to create an adsorbent, effectively transforming one waste stream into a solution for another, thereby significantly reducing costs. By repurposing textile factory waste, particularly AFW, as an efficient adsorbent, this material can effectively remove both basic and acidic dyes from textile dyeing and printing wastewater.

The modified AFW is non-porous, and the surface area is attributed to inter-particle voids (macropores). The pore size was reached to 23.814 nm. The modification of AFW with NaOH/ DMF has shown high loss in weight %, due to the effect of DMF.

The modified fibers demonstrated superior durability and efficiency compared to their unmodified counterparts. Specifically, the Na- ethoxide modified fiber waste achieved 88% MB removal in the first cycle and maintained a 47% removal efficiency after six cycles.

## Data Availability

We declare that the data used during the current study are manually generated inside the laboratory, and results are shared already in this study. However if extra information are needed, they are available, and the primary corresponding authors can share them with reasonable request. All data used in this study are generated inside the laboratory, and calculations are done manually. However, in table 4, we have compared our work with other published studies and we have cited all these studies.

## Code availability

We declare that the data used during the current study are manually generated inside the laboratory, and results are shared already in this study. However if extra information are needed, they are available, and the primary corresponding authors can share them with reasonable request.
